# Where and How Are Roads Endangering Mammals in Southeast Asia's Forests?

**DOI:** 10.1371/journal.pone.0115376

**Published:** 2014-12-18

**Authors:** Gopalasamy Reuben Clements, Antony J. Lynam, David Gaveau, Wei Lim Yap, Stanislav Lhota, Miriam Goosem, Susan Laurance, William F. Laurance

**Affiliations:** 1 Centre for Tropical Environmental and Sustainability Science and College of Marine and Environmental Sciences, James Cook University, Cairns, Queensland, Australia; 2 Kenyir Research Institute, Universiti Malaysia Terengganu, Kuala Terengganu, Malaysia; 3 Panthera, New York, New York, United States of America; 4 Rimba, 4 Jalan1/9D, Selangor, Malaysia; 5 School of Geography, University of Nottingham Malaysia Campus, Selangor, Malaysia; 6 School of Science, Monash University, Selangor, Malaysia; 7 Center for Global Conservation, Wildlife Conservation Society, New York, New York, United States of America; 8 Center for International Forestry Research, Bogor, Indonesia; 9 World Wide Fund for Nature-Malaysia, Jalan PJS 5/28, Petaling Jaya, Selangor, Malaysia; 10 Department of Husbandry and Ethology, Czech University of Life Sciences, Prague, Czech Republic; University of New England, Australia

## Abstract

Habitat destruction and overhunting are two major drivers of mammal population declines and extinctions in tropical forests. The construction of roads can be a catalyst for these two threats. In Southeast Asia, the impacts of roads on mammals have not been well-documented at a regional scale. Before evidence-based conservation strategies can be developed to minimize the threat of roads to endangered mammals within this region, we first need to locate *where* and *how* roads are contributing to the conversion of their habitats and illegal hunting in each country. We interviewed 36 experts involved in mammal research from seven Southeast Asian countries to identify roads that are contributing the most, in their opinion, to habitat conversion and illegal hunting. Our experts highlighted 16 existing and eight planned roads - these potentially threaten 21% of the 117 endangered terrestrial mammals in those countries. Apart from gathering qualitative evidence from the literature to assess their claims, we demonstrate how species-distribution models, satellite imagery and animal-sign surveys can be used to provide quantitative evidence of roads causing impacts by (1) cutting through habitats where endangered mammals are likely to occur, (2) intensifying forest conversion, and (3) contributing to illegal hunting and wildlife trade. To our knowledge, ours is the first study to identify specific roads threatening endangered mammals in Southeast Asia. Further through highlighting the impacts of roads, we propose 10 measures to limit road impacts in the region.

## Introduction

Habitat loss and overhunting are two major drivers of biodiversity declines, particularly for terrestrial mammals in tropical forests [1]–[3]. The expansion of roads can be a precursor to both of these threats [4]–[6]. Roads are proliferating across the planet at unprecedented rates and are increasingly seen as a severe environmental challenge [7]–[10]. Road development particularly jeopardizes conservation initiatives in developing countries, where increasing road densities are linked with economic growth and habitat degradation [11]. Between 2005 and 2010, the percentage of total roads that are paved in the developing countries of East Asia soared from 16% to 51% [12].

Roads have a number of direct, negative impacts on mammals. They can impede animal movements (thereby decreasing access to habitats and preventing gene flow; [13]), result in roadkills [14], [15], cause behavioural avoidance of traffic [16], [17] and roadside habitats [18] and promote elevated hunting pressure [19]. Over time, roads can also increase the susceptibility of mammal habitats to human colonization and exploitation [20], [21]. A review of 79 empirical studies demonstrated that roads have a net negative effect on animal abundance and species richness, particularly for large-bodied mammals [22]. In fact, population densities of sensitive mammal species can decline up to 5 km from linear infrastructure such as roads [23]. When the knock-on effects of habitat loss and fragmentation are considered, these distances become much greater.

Research conducted on the impacts of roads on mammals, and biodiversity in general, appears to have a geographic bias. The majority of such studies have been conducted outside of the tropics (≥76% of 244 published studies; [24]). In the tropics, negative impacts of roads on mammals have been documented in South America [25], Central Africa [19], [26] and Australia [27]. In Southeast Asia, where deforestation rates are the highest of the major tropical regions [28], studies explicitly investigating the impacts of roads on mammals are surprisingly scarce. Out of 533 road-related biodiversity studies identified in a keyword search in the BIOSIS Previews database (S1 Method), only one explicitly investigated the impacts of roads on mammals in Southeast Asia [29].

In Southeast Asia, between 21–48% of all native mammal species are predicted to be extinct by 2100 [30]. Major extinction drivers include forest conversion for agriculture and exotic-tree plantations [31], [32] and market-driven hunting for bush meat, valuable body parts and traditional medicine [33], [34]. Because roads strongly influence all these threats, knowledge about where and how roads are affecting endangered mammals in Southeast Asia is urgently needed to develop evidence-based mitigation measures. If such measures are to be successfully implemented, conservation practitioners must identify which roads are most likely to promote forest conversion, hunting and trade in the region. Where possible, empirical evidence of road impacts on endangered species should also be obtained, in order to develop appropriate mitigation measures.

Here, we present three lines of evidence concerning where and how roads are impacting endangered mammals and their habitats in Southeast Asia. First, we asked experts to identify roads that currently or potentially threaten endangered species through forest conversion and illegal hunting. Second, we gathered evidence from peer-reviewed articles and grey literature to assess the threats from each road and the presence of endangered species around them. Third, we developed detailed case studies based on species distribution models, satellite imagery and animal sign surveys to illustrate how roads (1) cut through habitats where endangered mammals are likely to occur, (2) have led to intensified forest conversion, and (3) contribute to illegal hunting and the wildlife trade. Based on these case findings, we highlight key lessons regarding road proliferation and propose strategies to minimise their negative impacts on endangered mammals in Southeast Asia.

## Materials and Methods

### Ethics statement

This study was conducted as part of GRC's Ph.D. thesis that received a human research ethics approval from the James Cook University Human Research Ethics Committee (No. H3655, The impacts of roads on large mammals and indigenous people in Southeast Asia, 31 Mar 2010 – 21 Feb 2012; category 1). This approval permitted interviews consisting of questions to obtain information on perceptions of roads and resource harvesting patterns along roads. Our questionnaire to experts explicitly guaranteed anonymity and completion of the online questionnaire itself implied consent. The questionnaire contains no identifying information linking it to the respondents.

### Where are roads threatening endangered mammal habitats?

Field workers may provide the best available information about roads threatening endangered mammals in the region. As expert interviews can be used to gain insight into contemporary biodiversity threats such as roads [35], we emailed brief questionnaires to experts in mammal research and/or conservation from relevant scientific institutes and universities, environmental NGOs and wildlife departments in the following countries and sub-regions: Cambodia, Lao PDR, Indonesia (Irian Jaya, Java, Sulawesi, Sumatra, Kalimantan), Malaysia (Peninsular Malaysia, Malaysian Borneo), Myanmar, Philippines, Thailand and Vietnam. At least one expert from each country and sub-region was contacted. To maximise response rates, each expert opinion was limited to a maximum of three roads believed to contribute to forest conversion and illegal hunting/trade. We requested road names and threatened mammal habitats. Several experts who did not respond in writing were subsequently interviewed by telephone. To minimize observer and organisation bias, only roads named by at least two respondents with different affiliations were highlighted. However these criteria were relaxed in Myanmar where there is a paucity of relevant experts working in the country. Respondents also identified proposed roads in their country. Proposed roads were included without bias reduction because potential roads may be insufficiently publicised for corroboration by different experts. Compiled information was returned to country experts for final verification. Lastly, we assessed expert claims of roads affecting endangered mammals with information from peer-reviewed articles and grey literature.

We acknowledge two caveats here. First, the list of roads identified by experts is not exhaustive for Southeast Asia, especially when respondents were limited in number – there could certainly be more important roads than were captured by our interviews. Second, the list of roads for each country does not necessarily represent the most threatening roads in terms of impact on endangered mammals, but are merely prominent *examples* based on the experience of experts working in each country.

### How are roads threatening endangered mammal habitats?

#### Do roads bisect habitats where endangered mammals are likely to still occur?

Expert claims of roads cutting through habitats where endangered mammals are likely to still occur should ideally be supported by empirical evidence. If data on the presence of species around roads are available, species-distribution models can be constructed to illustrate highly suitable habitats around the road and decide whether a planned road would cut through these important habitats.

Using Maximum Entropy (MaxEnt) models, we modelled presence-only data on the endangered Asian Tapir (*Tapirus indicus*) in Peninsular Malaysia for three roads identified by experts (S1 Table) to assess whether they pass through important habitats for this species. MaxEnt models examine the probability of occurrence in presence-only data as a function of environmental variables by randomly selecting background pixels as pseudo-absences [36]. When the three roads were laid over a MaxEnt-predicted distribution map for the Asian Tapir (see [37] for method), overlap with suitable tapir habitats was quantified (logistic values ≥0.5 indicate suitable habitats [38]).

Predictions by MaxEnt models, however, have certain weaknesses. They do not account for imperfect detections [39], and the indices are not directly related to probability of occurrence, a more informative measure of the importance of habitat [40].

When resources are available for a more in-depth quantification of important mammal habitats, detection/non-detection data obtained from surveys conducted under an occupancy framework [41] can be used to generate occupancy maps or habitat-use-intensity maps that account for imperfect detections. We obtained such data from camera-trapping surveys to generate habitat-use-intensity maps for the same species, the endangered Asian Tapir (S2 Method). The data were collected from two forest blocks (lower and upper) on either side of State Road 156, a road identified by one of the experts in Peninsular Malaysia (see [42] for survey methods). Subsequently, we calculated the mean habitat-use estimates of the Asian Tapir affected by the path of the road.

#### Does forest conversion intensify following road construction?

Freely available satellite images are useful for detecting changes in forest cover along roads. Landsat satellite imagery is ideal for this purpose because it is regularly acquired, has global coverage, medium spatial resolution (30–80 m) and large historical archives [43]. Although Landsat 7 images have issues with missing data, methods are available to ensure Landsat composites are comparable over considerable temporal scales [44]. From individual Landsat satellite images, false-colour composites can be created to differentiate roads from vegetation and bare or built-up areas. As an example, we created a composite for one road identified by experts in Cambodia, Provincial Road 76, which bisects the Snuol Wildlife Sanctuary, a protected area managed by the Cambodian Ministry of Environment (12° 5'26.98"N; 106°39'40.83"E). We chose this road due to the availability of actual observations on the ground by our coauthor AL.

When satellite imagery of a road is available over a period of time, further analysis can provide more detailed information on its impact on surrounding forests. Once the images are classified, an intensity analysis [45] can be performed (S3 Method) to examine before and after road construction: (1) the intensity of gains and losses in gross primary forest, forest mosaics and bare or built-up areas changes; (2) whether there are differences in annual rates of change in overall land categories; (3) whether primary forests were avoided or targeted by transitions to bare or built-up areas; and (4) whether forest conversion occurred close to or further from the road.

To obtain data for the intensity analysis, we classified land cover using georeferenced and orthorectified cloud-free Landsat 4, 5 (TM) and Landsat 7 (ETM+) images at 30-m resolution for the same road bisecting Snuol Wildlife Sanctuary. Analyses were run at three time intervals: when the road (1) was absent (1990), (2) was recently completed (2001), and (3) had existed for some time (2009). Using both the original satellite data and Google Earth images as auxiliary references, and information on forest types, classified data were manually defined and merged into 5 land-cover categories: 1) primary forest; 2) mosaic (i.e., secondary forest/regrowth/scrub); 3) bare or built-up areas; 4) other (i.e., riparian/swamps); and 5) water bodies.

To examine whether transitions of primary forest and mosaic to bare or built-up areas occurred close to or further from the road, we created kernel density plots. Kernel density plots were preferred over histograms for examining the distribution of the continuous variable “distance from road”, because kernel estimates converge more quickly to true underlying densities [46]. Land-cover classification was carried out using ENVI 4.8 (ITT, Boulder), cross-tabulation matrices were created in IDRISI Selva (Clark Labs, Worcester) and GIS analyses were performed in ArcMap 10.0 (ESRI, Redlands).

#### Do roads contribute to illegal hunting and wildlife trade?

When collected in a systematic manner, signs of camps and snares targeting mammals can be used to provide empirical evidence of roads contributing to illegal hunting. If mammals that are targeted by poachers are common near roads, we expect hunting signs to increase with increasing proximity to the road. Access for poachers is easier close to the road and it is more convenient to transfer hunted animal products to vehicles. We surveyed for illegal hunting signs in the same forest blocks mentioned earlier on either side of State Road 156 in Peninsular Malaysia (S1 Table). Three temporal replicates of sign surveys were carried out on foot during the dry season (May - Oct 2011). Surveys in each cell covered three habitat types (animal trail, ridge or old logging road) where detection probability of both large mammals and hunting signs are likely to be high. Among the three temporal replicates, route overlaps were minimised to achieve spatial independence and greater coverage within each cell. Kernel density plots were used to ascertain the distribution of hunting signs in relation to the road from 131 notionally independent survey routes.

Roads have also been implicated in the illegal trade of mammals and other wildlife [47], [48]. Myanmar has been recognised as a major illegal source of animal parts to consumer and re-export markets in China and Thailand [49]–[53]. Identifying trading routes is the first step to help suppress illegal trade, which is now a key priority for recovering the country's depleted tiger population [54]–[56]. With help from the Wildlife Conservation Society (WCS) Myanmar programme, we mapped indicative trading routes in the country, mainly using information from hunting and market surveys, interviews with villagers, police and township officials, and field survey data. Indicative trade routes were defined as the roads linking places where wildlife was reported to be sourced from forest areas, with places they were reported to be traded, usually markets in regional towns, and border areas. As the road network in Myanmar's rural areas has essentially been unchanged in the last 50 years, options for trafficking wildlife are limited to the main roads along which all vehicular traffic moves.

## Results and Discussion

### Location of roads threatening endangered mammal habitats

Local mammal experts identified 16 roads as locations threatening endangered mammal habitat. These roads occur in 10 of the 13 sub-regions in seven SE Asian countries (S1 Table). A total of 25 endangered mammal species (International Union for the Conservation of Nature categories EN and CR) have been reported to occur in the vicinity of roads identified by our experts – this is around 21% of the total number (117) of endangered terrestrial mammal species known to occur in the represented countries (S1 Table). In view of their potential threats, 8 proposed road construction or upgrading projects were also identified (S2 Table).

### Evidence of roads threatening endangered mammal habitats

#### Roads have cut through habitats where endangered mammals are likely to occur

**Figure 1:**
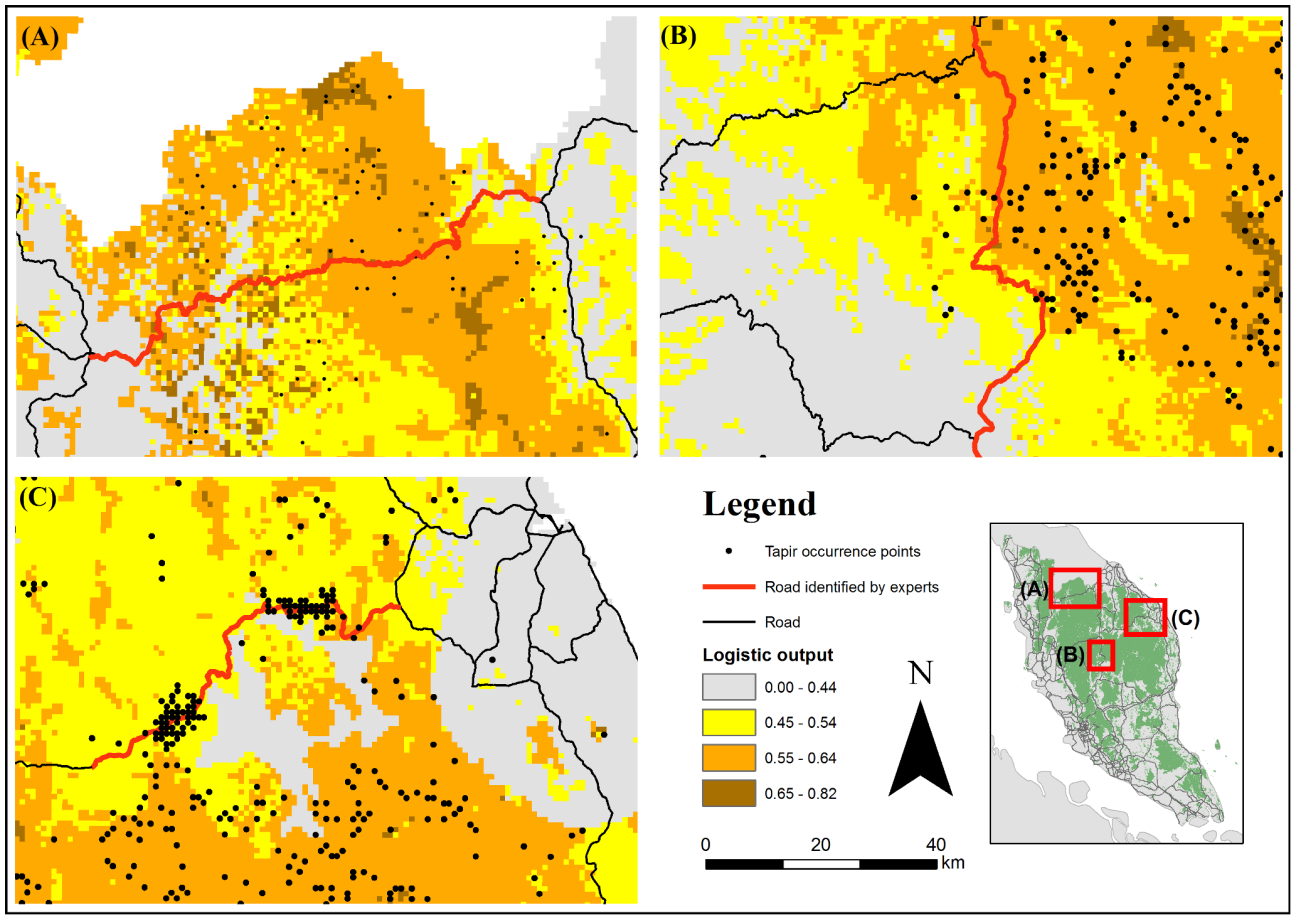
Habitat-suitability map for the endangered Asian Tapir (*Tapirus indicus*) generated by Maximum Entropy (MaxEnt) modelling. Three roads identified by experts in Peninsular Malaysia seem to cut through important habitats (pixels with logistic value ≥0.45) for this species: (A) Federal Route; (B) Federal Route 8; and (C) State Route T156.

Our results indicate that MaxEnt modelling can be a useful approach to investigate whether roads cut through endangered mammal habitats. All three roads identified by experts in Peninsular Malaysia cut through highly suitable habitats (logistic value ≥0.45) for the Asian Tapir, based on the mean (± SD) logistic value of pixels that all the roads passed through (Fig. 1): Federal Route 4 (0.50±0.13); Federal Route 8 (0.49±0.08); and State Route T156 (0.51±0.04). The MaxEnt-generated Asian Tapir habitat-suitability map had a mean (SD) AUC score of 0.76±0.02 [37], which is around the accepted potentially useful AUC score of 0.75 [57]. There is a clustering of presence-only points in State Route T156 due to intensive sampling, but this bias has been accounted for through the use of a bias grid (see [37]).

**Figure 2:**
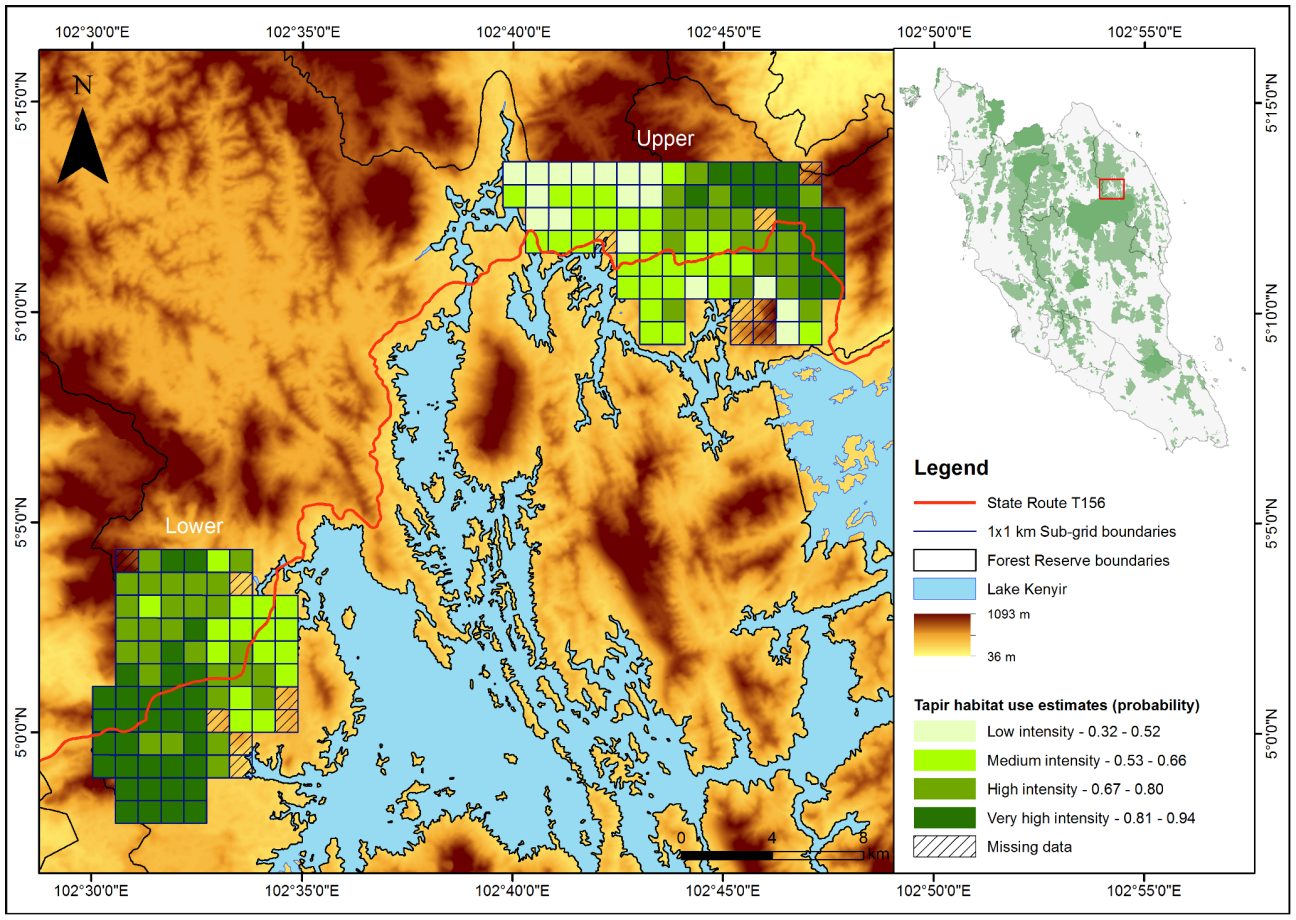
Habitat-use intensity map for the endangered Asian Tapir (*Tapirus indicus*). Habitats that are intensely used by this species appear to be bisected by State Route T156 in the State of Terengganu, Peninsular Malaysia.

Habitat-use intensity maps, which are more robust than maps derived from MaxEnt, indicated that State Route T156 also passes through forests that are intensely used by the Asian Tapir (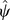 ± SE  = 0.75±0.07; Fig. 2). Effects of sampling and site covariates on habitat use are provided in S3 Table.

#### Forest conversion has intensified following road construction

**Figure 3:**
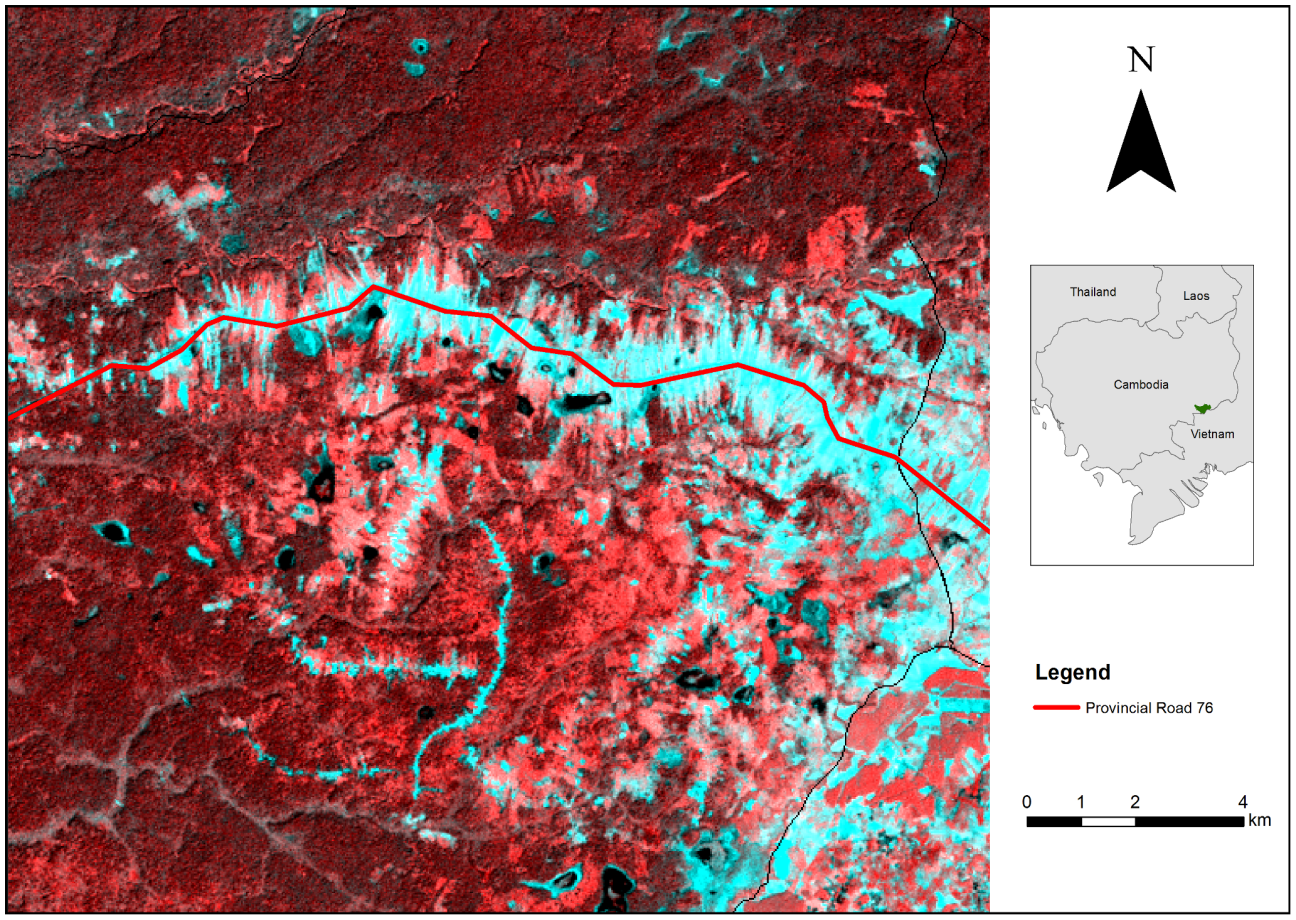
A false-colour composite of a Landsat 5 image over part of Snuol Wildlife Reserve, Cambodia. This technique reveals a ‘fish-bone’ pattern of arterial roads spawning from the larger Provincial Road 76 bisecting the wildlife reserve. Landsat Scene Path/Row: 127/52. Acquisition date: 09/12/2009.

A Landsat image illustrated how the construction of Provincial Road 76 consolidated urban development along itself. A false-colour composite of the same image differentiated vegetation from roads and bare or built-up areas (Fig. 3). A ‘fish-bone’ pattern of arterial roads spawning from the larger Provincial Road 76 was evident. This pattern is typically observed in landscapes where numerous lateral roads are facilitating forest conversion away from a main arterial road. These arterial roads have been physically verified by the WCS Cambodia programme, some of which were built to access newly granted Economic Land Concessions in the buffer zone of the adjacent Seima Protection Forest. According to WCS Cambodia, the roads were also associated with fire signals that were detected by MODIS imagery, indicating ongoing forest clearance.

**Figure 4:**
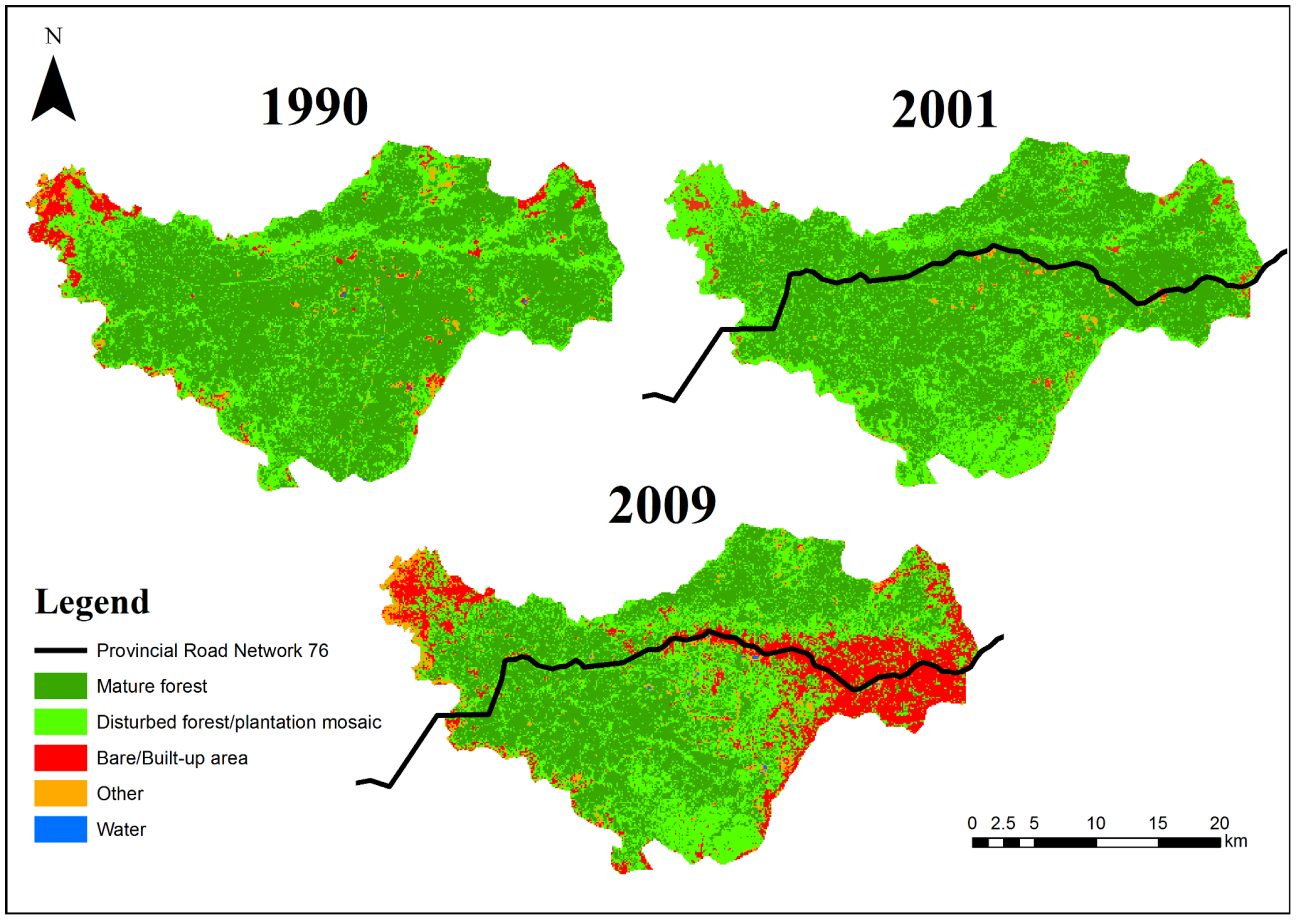
Land cover change of Snuol Wildlife Reserve. Cambodia. Landsat images were obtained for three time points: when the road was (1) absent (1990), (2) recently completed (2001), and (3) had existed for some time (2009). Inputs for land cover classification included the first three layers of a Tasseled-cap transformation (Kauth & Thomas 1976) and spectral bands 1–5 and 7. Data layers were processed using an unsupervised classification (ISODATA) algorithm with a maximum class of 200, and 50 maximum iterations with a convergence threshold of 0.95. Accuracy analysis was only conducted for the classified image from 2010 using the original Landsat 5 image and a Landsat 7 image from a comparable time period. The overall accuracy of the 2010 image was relatively high at 84.8%. The confusion matrix is provided in Table S4. Landsat Scene Path/Row: 127/52. Acquisition dates for Landsat 4, 5 (TM) and Landsat 7 (ETM+) images: 27/01/1990; 15/04/2001; and 09/12/2009.

**Figure 5:**
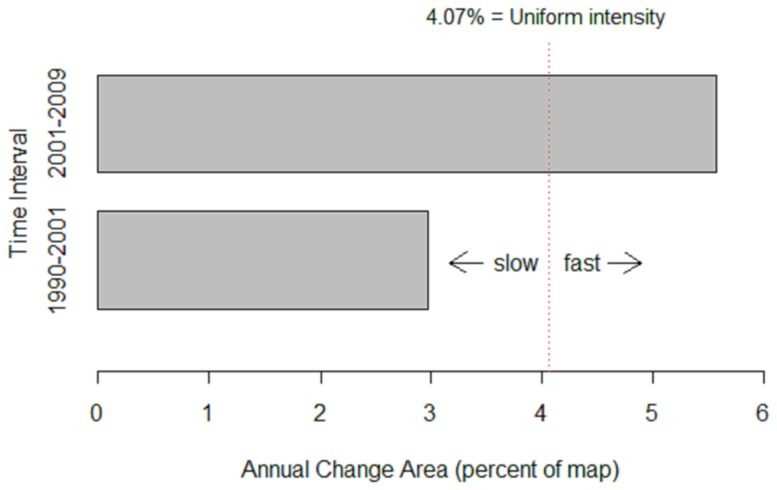
Time intensity analysis of land category change in Snuol Wildlife Reserve, Cambodia. Bars show intensity of annual area of change within each time interval: 1) 1990–2001 (road mostly absent) and; 2) 2001–2009 (road always present).

The intensity analysis of classified Landsat imagery also empirically demonstrated how the road has intensified forest conversion (Fig. 4; see S4 Table for confusion matrix). Calculations from three different years demonstrated that the observed gross gain in bare or built-up areas and gross loss of primary forest was greater in the 9 years between 2001 and 2009 when the road was always present than between 1990 and 2001, when the road was only operational for a short time near the end of the period. The annual rate of land category change in Snuol Wildlife Reserve also was much faster when the road was always present (Fig. 5).

The intensity analysis also provided three additional lines of evidence that forest degradation and loss intensified following the construction of the road through Snuol Wildlife Sanctuary. First, gains in mosaics (i.e., secondary forest/regrowth/scrub) and bare or built-up areas were more intense in the later interval than the earlier interval. Second, transitions to mosaics did not target primary forests in the earlier interval, whereas, in contrast, primary forests were altered to form mosaics when the road was always present (S5 Table). Third, although transitions to bare or built-up areas occurred more in mosaics in both time intervals, this happened at a much lower intensity in the earlier interval than later (S5 Table).

**Figure 6:**
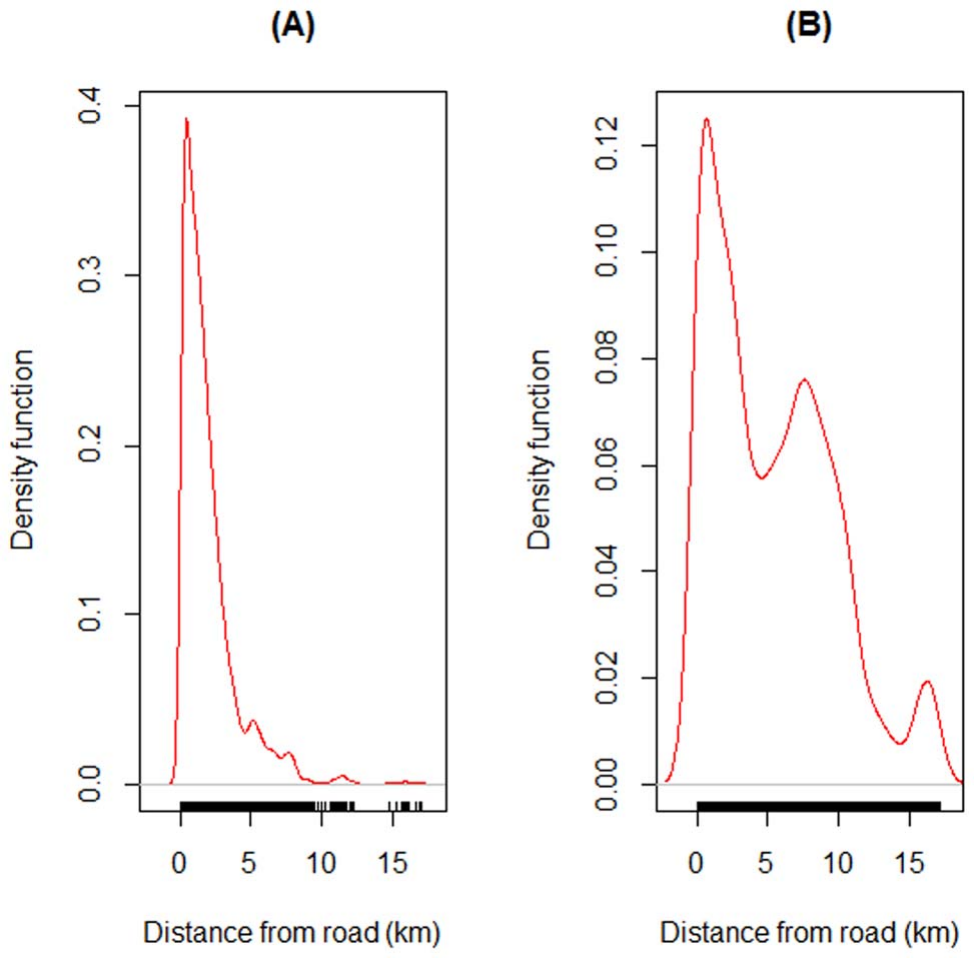
Kernel density plots of transitions of (A) primary forest and (B) secondary forest mosaics to bare or built-up areas in relation to distance from Provincial Road 76 bisecting the Snuol Wildlife Reserve, Cambodia.

Kernel density plots also indicated that the road through Snuol Wildlife Sanctuary probably contributed to forest conversion because most of the transitions of primary forest (Fig. 6A) and mosaics (Fig. 6B) to bare or built-up areas occurred closer to the road.

Expert claims of other roads facilitating forest conversion were largely corroborated by peer-reviewed articles and grey literature and we compiled the information in Table S6.

#### Roads have contributed to illegal hunting and trade of wildlife

**Figure 7:**
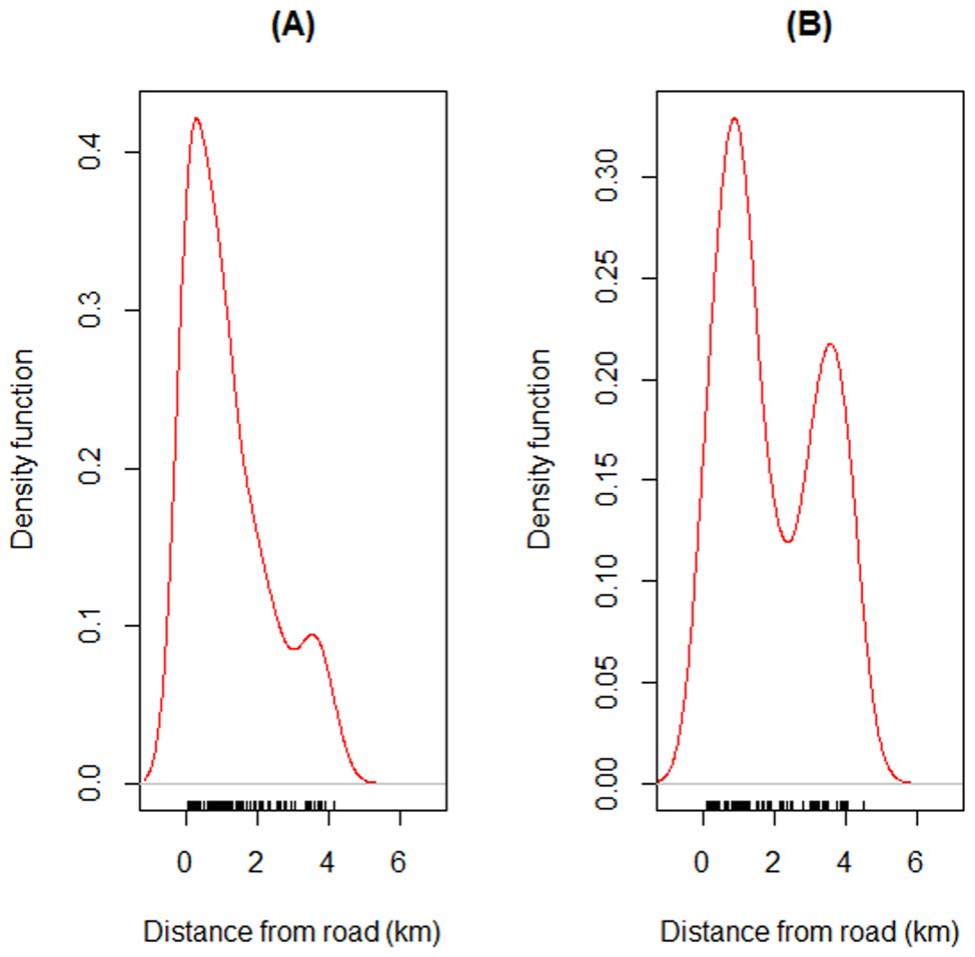
Kernel density plots of detections of (A) encroachment camps and (B) snares in relation to distance from State Route T156 cutting through forests in the State of Terengganu, Peninsular Malaysia.

At State Route T156 in Peninsular Malaysia, our indirect sign surveys recorded a total of 125 encroachment camps and 131 snares in the forests on either side of the road. Almost all hunting signs were illegal and of foreign origin. Kernel density plots revealed that detections of camps (Fig. 7A) and snares (Fig. 7B) were higher nearer to the road than further from it. In total, we recorded at least 43 access trails leading from the road into the forest.

**Figure 8:**
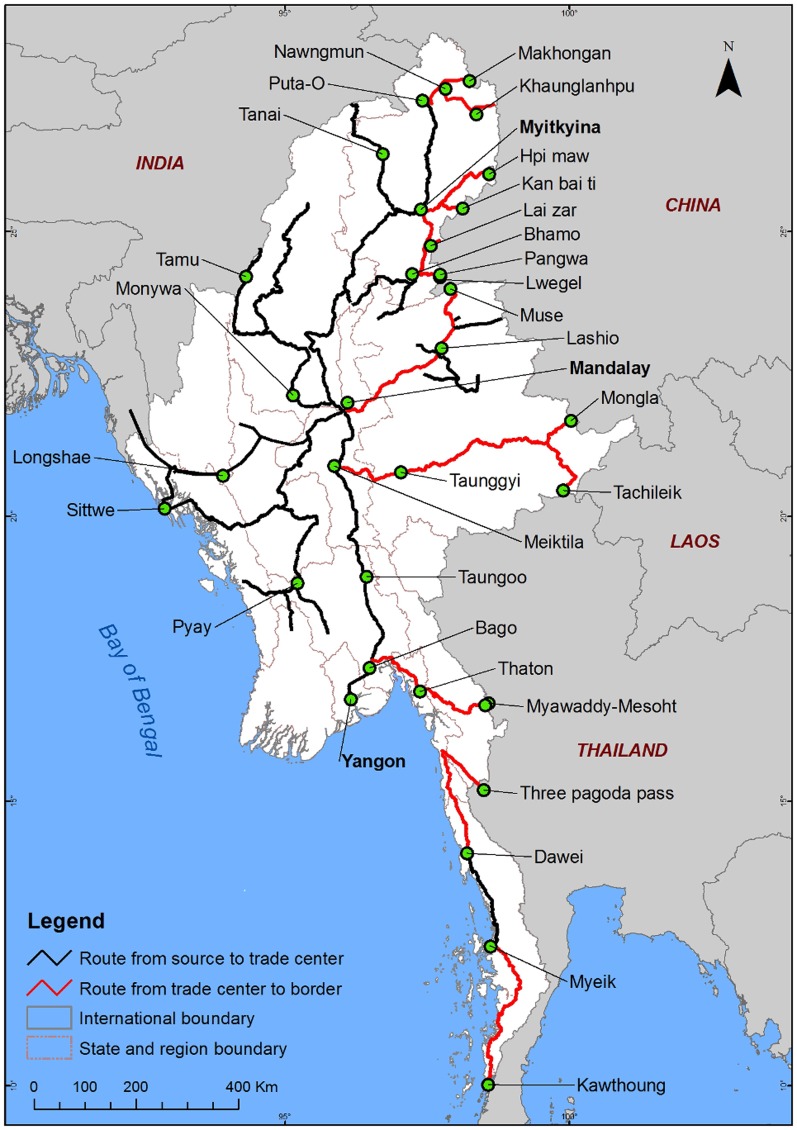
Map of road networks in Myanmar functioning as conduits for the illegal wildlife trade to border towns (circles) in neighbouring countries.

In Myanmar, information from the WCS Myanmar programme indicated that road networks are facilitating illegal trade of mammals at a national level. Specifically, routes from sources to trade centres, and trade centres to borders, were identified (Fig. 8). At the Thai-Myanmar border, parts of at least 187 bears and 1158 felids were recorded between 1999 and 2006 at border markets such as Three Pagoda Pass and Tachilek ([52], [58]; Fig. 8). Improved road links across the border and upgraded highways, such as those connecting Mandalay, Lashio and Muse cities (Fig. 8), appear to have increased access for traders to lucrative border markets in China [51].

Expert claims of other roads facilitating illegal hunting and trade were largely corroborated by peer-reviewed articles and grey literature and we compiled the information in S7 Table.

### Lessons learnt from road proliferation in Southeast Asia

To our knowledge, this is the first study to identify roads that are likely to threaten endangered mammals and their habitats in Southeast Asia. Most of the specific roads identified by experts bisect or are adjacent to national parks and to a lesser extent, production forest reserves. Below, we summarize some general lessons from road proliferation in this region.

#### Drivers of road construction

Roads in this region are not always built to benefit rural society, as is often claimed. While the expansion of road infrastructure has alleviated poverty in many countries [59], surveys in Lao PDR revealed that the poorest rural residents ranked the value of roads or access to markets as only 8^th^ out of 12 potential measures to improve their income levels [60]. Their income levels are typically too low to afford the supplies that roads bring into their areas [61].

Road development projects are sometimes initiated with hidden agendas. In Lao PDR, almost two-thirds of timber supplies over the last five years have come from clearances associated with development projects that include road construction [62]. In Sumatra, the Governor of Aceh pushed for more proposed roads through the Leuser ecosystem under the expanded Ladia Galaska road scheme. The roads probably benefited local communities to some extent, especially by decreasing transportation time of timber and agricultural commodities and to free enclaved villages from isolation [4]. However, critics argue that financial benefits would only be reaped by security forces and local elites from illicit business opportunities [63], rather than roads providing a net benefit to local communities [64], [65].

Socio-political factors also pose a serious challenge for opposition to roads on environmental grounds. For example, the Ladia Galaska road scheme is largely supported by the Achenese people, not only because it would greatly improve intra-provincial transport efficiency (especially for agricultural commodities such as palm oil; [4]), but also because they would be less reliant on roads going through neighbouring provinces (M. Linkie, personal communication).

Ultimately, government financial capacities may determine whether a road threatens biodiversity. In Vietnam, the Ho Chi Minh Highway is now regarded as the ‘single largest long-term threat to biodiversity’ in the country [66]. Before its construction, an option of diverting it around Vietnam's oldest national park was rejected by the government to avoid costs of $20 million to resettle 900 households [67]. Under rare circumstances, an economic crisis might even help abate the impacts of roads on biodiversity. During the financial crisis in 1998, for example, the Indonesian government cut back on funds for the construction and maintenance of major highways, causing delays of up to seven years in some road projects in Kalimantan [68].

#### Road impacts vary

Logging road network density can influence the degree to which logging impacts biodiversity. For example, the impacts of logging on biodiversity in parts of Southeast Asia has generally been more severe than that in the Amazon, where selective logging typically occurs at a low intensity and roads are usually less dense [25]; but see [69]). In Borneo, satellite images revealed 271,819 km of large (>15 m wide) logging roads were built between 1973 and 2010 [70]. While many logging roads experience forest re-growth after logging, logging activities have usually resulted in forest conversion throughout most of Borneo, especially in Kalimantan [71]. By increasing forest access and creating much dry, flammable slash, logging activities and roads also appear to have increased forest fires; 76% of 258 fire-prone zones in Kalimantan contained logging roads [72].

Roads have also been known to contribute to forest conversion at a distance. In East Kalimantan, to escape detection from police and forestry officials, people migrated away from the Balikpapan-Samarinda Road into the Bukit Soeharto Recreation Forest to illegally clear land for pepper plantations [73].

In rare instances, road development may even be used as a wildlife conservation strategy. In Vietnam, the widening of a road near Cat Tien National Park was deemed an appropriate measure to discourage elephant movement to areas where they could potentially be killed in human-dominated landscapes [74].

### 10 ways to mitigate impacts from road development

We suggest 10 measures to minimise the negative impacts of road development in and around endangered mammal habitats in the region.

#### (1) Maintain and improve forest connectivity on either side of existing roads

The integration of green infrastructure options (e.g., underpasses, overpasses, road signs and culverts) into proposed road designs, along with incorporating measures to evaluate their efficiency of use may be beneficial for the movement of mammals through fragmented habitats [75]–[78]. In Cambodia, the preservation of forests on both sides of Provincial Road 48 and 76 was highlighted as a key strategy [79] to ensure the dispersal of arboreal species such as the Yellow-cheeked Crested Gibbon (*Nomascus gabriellae*).

#### (2) Increase enforcement effort along existing roads through endangered species habitats

Our study has indicated locations along trade routes in Myanmar where additional law enforcement effort is urgently required (Figure 8). Elsewhere in the region, the need to increase enforcement efforts along roads to deter illegal hunting has already been recognized in some countries. Along Federal Route 4 in Peninsular Malaysia (S1 Table; Fig. 1), government enforcement agencies stepped up enforcement efforts after World Wide Fund for Nature (WWF)-Malaysia patrols detected large number of snares along roadside forests (A. Zafir, pers. comm.). In Lao PDR, staff of protected areas blame roads in general for increased hunting by locals, foreigners and road construction crews [61]. As such, road check points along Route 1C, which bisects the Nam Et-Phou Louey National Biodiversity Conservation Area, have been recommended by the Wildlife Conservation Society (WCS) as a vital measure to curb tiger poaching and the illicit trade in ungulate prey species from core tiger conservation areas [80]. A provincial forest law enforcement strategy also identified closing illegal roads as a required action for reducing forest encroachment and illegal timber extraction [81].

#### (3) Minimise threats from logging roads via sustainable forest management regimes

Closing logging roads after cessation of logging can help restrict access to poachers and illegal loggers [82]–[85]. Forestry departments should prioritise the closure of logging roads that contribute to the transport of illegally harvested timber. This is especially important at the Malaysian-Indonesian boundary on Borneo where, on satellite images, 137 cross-border logging road intrusions have been detected [86]. When new logging roads are constructed through previously undisturbed mammal habitats, greater law enforcement must be afforded for newly accessible resources [62], together with publicised policies and measures that deter poaching [76].

#### (4) Resolve land rights and tenure prior to road construction

One of the key drivers of habitat loss is the absence of land and resource tenure along roads. This has resulted in an uncontrolled influx of locals seeking to clear and claim land along roads [87]. To minimise illegal settlements along roads bordering important biodiversity areas, relevant government agencies should complete the allocation of lands for villages and protected areas prior to road construction.

#### (5) Increase engagement with road development agencies in conservation planning

Agencies responsible for road development are rarely included as main project partners in species conservation plans (e.g., [88], [89]). Because roads can be the precursor of forest conversion and hunting, road-relevant stakeholders should be included in the early stages of conservation planning. Plans should include scientifically-sound guidelines of where roads can be constructed or upgraded to maximize agricultural benefits and minimize biodiversity loss [10]. In Sumatra, timely discussions with villagers and local government officials prevented a road from cutting through Bukit Barisan Selatan National Park [90]. In the long run, such engagements can facilitate greater transparency and improved lines of communication between protected area managers and road authorities. Such communication gaps are common in countries such as Lao PDR, where heads of protected areas are rarely consulted before nearby roads are constructed [61]. It is unsurprising that state government infrastructure projects are one of the key drivers of deforestation in northern Lao PDR [91].

#### (6) Integrate road planning across relevant government agencies

Ad hoc planning with little or no cross-sectoral communication between governmental departments is often the root of environmental problems associated with roads. Encouragingly, in Lao PDR, an Environment Unit has been created within the Department of Roads to ensure environmental concerns are considered in road construction programmes [62]. In Malaysia, the Department of Wildlife and National Parks laudably worked together with the Public Works Department to incorporate underpasses along a new highway to facilitate mammal migration in important wildlife corridors [92]. However, multi-agency road planning must take place at appropriate government levels. For instance, conservation and development plans in Lao PDR should be developed at provincial rather than local levels as most threats to protected areas, especially road construction, are likely to originate from the former [62], [81].

#### (7) Conduct projections of economic and biodiversity loss prior to road development

**Figure 9:**
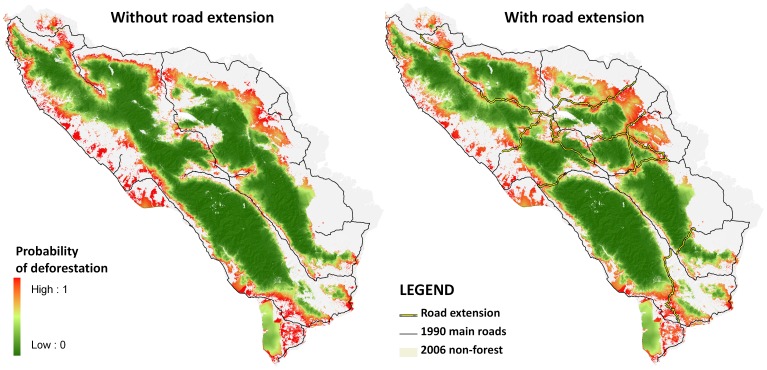
Probability map of deforestation (A) without further Ladia Galaska road extension, and (B) with road extension. Source: [4].

Predictions of how road development will result in biodiversity and economic loss may help guide decision-making involving road planning. In Sumatra, the government plans to expand the Ladia Galaska road scheme, an all-weather road network in Aceh. However, it is feared that this road development will further reduce and fragment mammal populations [93], especially two of the three largest remaining Orangutan populations [94]. Indeed, a study projected that the total economic value of the Leuser ecosystem under selective use would be greater than a 30-year deforestation scenario [65], which would certainly be realized under an expanded Ladia Galaska road scheme cutting through the protected area. Predictive models have also shown that forest areas near roads in Aceh are highly vulnerable to deforestation, with areas at high risk of deforestation (*p*>0.8) predicted to increase by 40% (Fig. 9). In fact, Orangutan habitat is predicted to further decline by 16% (1,137 km^2^) in 2030, resulting in the loss of an estimated 1,384 individuals (or 25% of the current global population; [4]).

#### (8) Explore compensation schemes that can minimise the need for, or impact of proposed roads

Inter-governmental REDD (Reduced Emissions from Deforestation and Degradation) projects, such as the recent Norway-Indonesia pact [95], may have prevented the construction of new logging roads through peat swamps and natural forests. Another possible strategy is the implementation of carbon-deposit and refund systems by financial lending institutions [96]. Under this mechanism, a road developer is obliged to buy credits equal to the net carbon emissions expected from deforestation along an existing or proposed road. These credits then serve as deposits over fixed periods. At the end of each period, the road developer is allowed to sell credits equal to the difference between expected and actual deforestation – this means the developer would redeem all deposits on all the forest maintained intact and retire remaining credits to cover deforestation that actually occurred. According to Reid [96], one advantage for the developer is that there is a conservation incentive beyond the construction phase to avoid all deforestation because forest conversion, as we have shown, can intensify after a road is built. If the developer has taken steps to minimise deforestation along the road, the developer will financially benefit once market prices for carbon have risen. However, if the present value of carbon does not increase considerably, the uptake of this scheme will remain slow due to the high uncertainty of reaping attractive financial gains. Further, a conflict in the underlying principle of this REDD scheme needs to be resolved because increasing amounts of carbon will be emitted in the long run from vehicles using the road.

Ultimately, financing governments should conduct due-diligence studies prior to a road project overseas. In Lao PDR, for example, it was unlikely that the Australian Government was aware of the potential environmental consequences from the rehabilitation project along Route 9 [97]. If a road must be built, offset mechanisms should be explored such as Payment for Ecosystem Services (PES). In Lao PDR, the economic value of an area in Nakai-Nam Theun Protected Area that was inundated by a hydro-electric dam project was offset by a contribution of US$31.5 million to create a management authority [76]. However, the effectiveness of these funds has come under intense scrutiny from both conservation and development agencies (AJ Lynam pers. obs.).

#### (9) Audit environmental and social impact assessments

Regional and global impact assessments that consider biological, social and economic trade-offs should be conducted for major roads and highways. Similarly, smaller roads at local scales should not be spared from assessments even if funds are constrained [62]. Unfortunately, impact assessments for forest clearance projects are not mandatory, and are mostly weak in Southeast Asia [76], while negative impacts of road construction highlighted in impact assessments rarely deter projects from going ahead. For example, most of the proposed roads in the Ladia Galaska scheme have not undergone Environmental Impact Assessments (EIAs), and those that did flouted regulations nonetheless [64]. In Lao PDR, the upgrade of Route 3 proceeded even after warnings from consultants about the negative impacts of the road construction [98].

#### (10) Raise public awareness of environmental impacts of road projects

In Kalimantan, roadside campaigns to raise awareness of fire prevention and suppression [99] indirectly helped to prevent further loss of fire-prone mammal habitats. In Peninsular Malaysia, increasing media attention given to the poaching issues along Federal Route 4 [100], [101] helped galvanise more support from enforcement agencies [102]. In Sumatra, media campaigns by NGOs convinced donor agencies such as the World Bank to discontinue financial assistance to the Indonesian state budget to prevent misuse of funds in road expansion projects such as the Ladia Galaska road scheme [103]. However, heightened awareness may not always reap immediate dividends. Banks continue to finance road projects in the Greater Mekong sub-region even though their own evaluation reports acknowledge that transnational roads contribute to human and wildlife trafficking [104].

## Conclusions

With the help of experts, we now know where several existing and planned roads are endangering mammals in Southeast Asia. However, greater effort should be expended to empirically elucidate the impacts of other potential roads using our recommended techniques. Implementing our suggestions for mitigation can help reduce the impacts of the roads highlighted by our experts. However, the implementation of these strategies can sometimes yield mixed results (S1 Box). Nevertheless, there are examples where roads have been rerouted in regions such as Kalimantan and Sumatra [90], [105]. This precedent gives us optimism that the impacts of roads on endangered mammals elsewhere in Southeast Asia can be ameliorated with sufficient awareness and political will.

## Supporting Information

S1 Table **Summary of 16 existing roads contributing to forest conversion of mammal habitats and hunting of endangered mammals according to 36 experts from seven countries in Southeast Asia (number of experts who responded/number of experts contacted).**
(DOCX)

S2 Table **Summary of 8 planned road construction or improvement projects that can potentially contribute to forest conversion of mammal habitats and hunting of endangered mammals according to experts from five Southeast Asian countries.**
(DOCX)

S3 Table **Logistic regression models examining the effect of four site covariates on the endangered Asian Tapir (** ***Tapirus indicus*** **) habitat use (** ***ψ*** **), and three sampling covariates affecting its detection probability (** ***p*** **), based on camera-trap data from forests along State Road 156, a road identified by one of the experts in Peninsular Malaysia.**
(DOCX)

S4 Table **Confusion matrix used in accuracy analysis of 2010 classified image from Snuol Wildlife Reserve, Cambodia.**
(DOCX)

S5 Table **Summary statistics for transition of land categories to mosaic of secondary forests from 1990–2001 and 2001–2009, and transition of categories to bare or built-up areas in 1990–2001and 2001–2009 in Snuol Wildlife Reserve, Cambodia.** Each row respectively gives: a) land category name, b) area of transition in terms of cell counts, c) intensity of transition per gross gain, d) uniform distribution of transitions across the area possible for that change, given the empirical gross gain for mosaic or bare or built-up areas, e) hypothesized uniform annual transition, f) annual number of pixels of hypothesized error, g) commission or omission intensity in t map and h) hypothesized error as percent of t map.
(DOCX)

S6 Table **Supporting evidence from peer-reviewed articles and grey literature corroborating expert claims that roads contribute to forest conversion of habitats where endangered mammals occur in Southeast Asia.**
(DOCX)

S7 Table **Supporting evidence from peer-reviewed articles and grey literature corroborating expert claims that roads contribute to illegal hunting and trade of wildlife in Southeast Asia.**
(DOCX)

S1 Method **Hierarchically-nested combinations of relevant keywords and wildcards used to search for road-specific biodiversity studies in Southeast Asia in the BIOSIS Previews database between 1985 and 2011.**
(DOCX)

S2 Method **Method used to generate habitat-use-intensity maps for the Asian Tapir (** ***Tapirus indicus*** **) from forests on either side of State Road 156, a road identified by one of the experts in Peninsular Malaysia.**
(DOCX)

S3 Method **Method for intensity analysis used to investigate whether forest conversion intensified following road construction in Snuol Wildlife Reserve, Cambodia.**
(DOCX)

S1 Box **Case study of real-world challenges faced during the implementation of recommended road impact mitigation measures in Kalimantan, Indonesia.**
(DOCX)

## References

[1] Brooks TM, Mittermeier RA, Mittermeier CG, da Fonseca GAB, Rylands AB, et al (2000) Habitat loss and extinction in the hotspots of biodiversity. Conserv Biol 16:909–923.

[2] Linkie M, Martyr DJ, Holden J, Yanuar A, Hartana AT, et al (2003) Habitat destruction and poaching threaten the Sumatran tiger in Kerenci Seblat National Park, Sumatra. Oryx 37:41–48.

[3] Chapron G, Miquelle DG, Lambert A, Goodrich JM, Legendre S, et al (2008) The impact on tigers of poaching versus prey depletion. J Appl Ecol 45:1667–1674.

[4] Gaveau DLA, Wich S, Epting J, Juhn D, Kanninen M, et al (2009) The future of forests and Orangutans (*Pongo abelii*) in Sumatra: predicting impacts of oil palm plantations, road construction, and mechanisms for reducing carbon emissions from deforestation. Environ Res Lett 4:034013.

[5] Suárez E, Morales M, Cueva R, Utreras Bucheli V, Zapata-Rios G, et al (2009) Oil industry, wild meat trade and roads: Indirect effects of oil extraction activities in a protected area in north-eastern Ecuador. Anim Conserv 12:364–373.

[6] Peh KS-H, Soh MCK, Sodhi NS, Laurance WF, Ong DJ, et al (2011) Up in the clouds: Is sustainable use of tropical montane cloud forests possible in Malaysia? BioScience 61:27–38.

[7] Laurance WF, Cochrane MA, Bergen S, Fearnside PM, Delamonica P, et al (2001) The future of the Brazilian Amazon. Science 291:438–439. 11228139 10.1126/science.291.5503.438

[8] Blake S, Strindberg S, Boudjan P, Makombo C, Bila-Isia I, et al (2007) Forest elephant crisis in the Congo Basin. PLOS Biology 5:e111. 17407383 10.1371/journal.pbio.0050111 PMC1845159

[9] Laurance WF, Balmford A (2013) A global map for road building. Nature 495:308–309. 23518547 10.1038/495308a

[10] Laurance WF, Clements GR, Sloan SP, O′Connell CO, Mueller NP, et al (2014) A global strategy for road building. Nature 513:229–232. 25162528 10.1038/nature13717

[11] Wilkie D, Shaw E, Rotberg F, Morelli G, Auzel P (2000) Roads, development, and conservation in the Congo Basin. Conserv Biol 14:1614–1622. 10.1111/j.1523-1739.2000.99102.x 35701921

[12] World Bank (2013) Data: Roads, Paved (% of total road). Available: http://data.worldbank.org/indicator/IS.ROD.PAVE.ZS. Accessed 2014 Aug 3.

[13] Lesbarrères D, Fahrig L (2012) Measures to reduce population fragmentation by roads: What has worked and how do we know? Trends Ecol Evol 27:374–380. 22356922 10.1016/j.tree.2012.01.015

[14] Goosem MW (1997) Internal fragmentation: the effects of roads, highways and powerline clearings on movements and mortality of rainforest vertebrates. In Laurance WF, Bierregaard ROJr, editors. Tropical forest remnants: Ecology, management and conservation of fragmented communities. Chicago: University of Chicago Press. pp.241–255.

[15] Colón CP (2002) Ranging behaviour and activity of the Malay civet (*Viverra tangalunga*) in a logged and an unlogged forest in Danum Valley, East Malaysia, J Zool. 257:473–485.

[16] Gubbi S, Poornesha HC, Madhusudan MD (2012) Impact of vehicular traffic on the use of highway edges by large mammals in a South Indian wildlife reserve, Curr Sci. 102:1047–1051.

[17] Vidya TNC, Thuppil V (2010) Immediate behavioural responses of humans and Asian elephants in context of road traffic in southern India. Biol Conserv 143:1891–1900.

[18] Roger E, Laffan SW, Ramp D (2011) Road impacts a tipping point for wildlife populations in threatened landscapes. Popuk Ecol 53:215–227.

[19] Blake S, Deem SL, Strindberg S, Maisels F, Momont L, et al (2008) Roadless wilderness area determines forest elephant movements in the Congo Basin. PLoS ONE 3:e3546. 18958284 10.1371/journal.pone.0003546 PMC2570334

[20] Laurance WF, Goosem M, Laurance SGW (2009) Impacts of roads and linear clearings on tropical forests. Trends Ecol Evol 24:659–669. 19748151 10.1016/j.tree.2009.06.009

[21] Vanthomme H, Kolowski J, Korte L, Alonso A (2013) Distribution of a community of mammals in relation to roads and other human disturbances in Gabon, central Africa. Conserv Biol 27:281–291. 23410077 10.1111/cobi.12017 PMC3644169

[22] Fahrig L, Rytwinski T (2009) Effects of roads on animal abundance: an empirical review and synthesis. Ecol Soc 14:21.

[23] Benítez-López A, Alkemade R, Verweij PA (2010) The impacts of roads and other infrastructure on mammal and bird populations: a meta-analysis. Biol Conserv 143:1307–1316.

[24] Taylor BD, Goldingay RL (2010) Roads and wildlife: impacts, mitigation and implications for wildlife management in Australia. Wildlife Research 37:320–331.

[25] Nepstad D, Carvalho G, Barros AC, Alencar A, Capobianco JP, et al (2001) Road paving, fire regime feedbacks, and the future of Amazon forests. Forest Ecol Manag 154:395–407.

[26] Laurance WF, Croes BM, Tchignoumba L, Lahm SA, Alfonso A, et al (2006) Impacts of roads and hunting on Central African rainforest mammals, Conserv Biol. 20:1251–1261. 10.1111/j.1523-1739.2006.00420.x 16922241

[27] Goosem M (2001) Effects of tropical rainforest roads on small mammals: Inhibition of crossing movements. Wildlife Res 28:351–364.

[28] Sodhi NS, Koh LP, Brook BW, Ng PKL (2004) Southeast Asian biodiversity: The impending disaster. Trends Ecol Evol 19:654–660. 16701328 10.1016/j.tree.2004.09.006

[29] Austin SC, Tewes ME, Grassman Jr. LI, Silvy NJ (2007) Road ecology of the leopard cat *Prionailurus bengalensis* in Khao Yai National Park, Thailand. Acta Zool Sinica 53:373–377.

[30] Brook BW, Sodhi NS, Ng PKL (2003) Catastrophic extinctions follow deforestation in Singapore. Nature 424:420–423. 12879068 10.1038/nature01795

[31] Koh LP, Wilcove D (2008) Is oil palm agriculture really destroying tropical biodiversity? Conserv Lett 1:60–64.

[32] Aziz SA, Laurance W, Clements R (2010) Forests reserved for rubber? Front Ecol Environ 8:178.

[33] Bennett EL, Robinson JG (2008) Hunting of wildlife in tropical forests: Implications for biodiversity and forest peoples. Washington DC: World Bank. 42 p.

[34] Bennett EL (2011) Another inconvenient truth: The failure of enforcement systems to save charismatic species. Oryx 45:476–479.

[35] Laurance WF, Useche DC, Rendeiro J, Kalka M, Bradshaw C, et al (2012) Averting biodiversity collapse in tropical protected areas. Nature 489:290–294. 22832582 10.1038/nature11318

[36] Phillips SJ, Anderson RP, Schapire RE (2006) Maximum entropy modelling of species geographic distributions. Ecol Model 190:231–259.

[37] Clements GR, Rayan DM, Aziz SA, Kawanishi K, Traeholt C, et al (2012) Predicting the distribution of the Asian tapir (*Tapirus indicus*) in Peninsular Malaysia using maximum entropy modelling. Integr Zool 7:402–409. 10.1111/j.1749-4877.2012.00314.x 23253371

[38] Elith J, Phillips SJ, Hastie T, Dudík M, Chee YE, et al (2011) A statistical explanation of MaxEnt for ecologists. Divers Distrib 17:43–57.

[39] Karanth K, Nichols JD, Hines JE, Karanth KU, Christensen NL (2009) Patterns and determinants of mammal species occurrence in India. J Appl Ecol 46:1189–1200.

[40] Royle JA, Chandler RB, Yakulic C, Nichols JD (2012) Likelihood analyses of species occurrence probability from presence-only data for modeling species distributions. Method Ecol Evol 3:545–552.

[41] MacKenzie DI, Nichols JD, Royle JA, Pollock KH, Bailey LL, et al**.** (2006) Occupancy estimation and modeling: Inferring patterns and dynamics of species occurrence. New York: Academic Press. 324 p.

[42] Clements GR (2013) The environmental and social impacts of roads in Southeast Asia. Ph.D. Thesis, James Cook University. Available: http://researchonline.jcu.edu.au/31888/. Accessed 2014 Aug 3.

[43] Wulder MA, White JC, Masek JG, Dwyer J, Roy DP (2011) Continuity of Landsat observations: Short term considerations. Remote Sens Environ 115:747–751.

[44] Wijedasa L, Michelakis DG, Sloan S, Clements GR (2012) Overcoming limitations with Landsat imagery for mapping of peat swamp forests in Sundaland. Remote Sens 4:2595–2618.

[45] Aldwaik SZ, Pontius Jr. RG (2012) Intensity analysis to unify measurements of size and stationarity of land use changes by interval, category and transition. Landscape Urban Plan 106:103–114.

[46] Scott DW (1979) On optimal and data-based histograms. Biometrika 66:605–610.

[47] Long B, Hoang M (2007) Recent records of and notes on the conservation of small carnivores in Quang Nam province, Central Vietnam. Small Carniv Conserv 34 &35:39–46.

[48] Shepherd CR, Compton J, Warne S (2007) Transport Infrastructure and Wildlife Trade Conduits in the GMS: Regulating Illegal and Unsustainable Wildlife Trade. Available: http://www.traffic.org/non-traffic/bci-paper.pdf. Accessed 2014 Aug 3.

[49] Martin EB, Redford T (2000) Wildlife for sale. Biologist 47:27–30. 11190214

[50] World Bank (2005) Going, going, gone: The illegal trade in wildlife in East and Southeast Asia. Washington DC: Environment and Social Development Department, East Asia and Pacific Region. 32 p.

[51] Shepherd C, Nijman V (2007) The trade in bear parts from Myanmar: An illustration of the ineffectiveness of enforcement of international wildlife trade regulations. Biodivers Conserv 17:35–42.

[52] Shepherd C, Nijman V (2008) The wild cat trade in Myanmar. Petaling Jaya: TRAFFIC Southeast Asia. 16 p.

[53] Oswell A (2010) The big cat trade in Myanmar and Thailand. Bangkok: TRAFFIC Southeast Asia. 32 p.

[54] Lynam AJ, Rabinowitz A, Khaing ST (1999) Tiger traces. Wildlife Conserv 102:36–41.

[55] Lynam AJ (2003) A national tiger action plan for the Union of Myanmar. New York: Myanmar Forest Department and the Wildlife Conservation Society. 58 p.

[56] Lynam AJ, Saw TK, Khin MZ (2006) Developing a national tiger action plan for the Union of Myanmar. Environ Manag 37:30–39. 10.1007/s00267-004-0273-9 16362487

[57] Phillips SJ, Dudík M (2008) Modeling of species distributions with MaxEnt: new extensions and a comprehensive evaluation. Ecography 31:161–175.

[58] Zaw M (2005) Open borders, demand keep wildlife trade going. Inter Press Service News Agency. Available: http://www.ipsnews.net/2005/05/burma-china-open-borders-demand-keep-wildlife-trade-going. Accessed 2014 Aug 3.

[59] Jones S (2006) Infrastructure challenges in East and South Asia. IDS Bull-I Dev Stud 37:29–44.

[60] Government of Laos (2000) An overview of quantitative and participatory poverty studies. Vientiane: State Planning Committee, Government of Laos.

[61] Robichaud R, Marsh CW, Southammakoth S, Khounthikoummane S (2001) Review of the national protected area system for Lao PDR. Vientiane: Lao-Swedish Forestry Programme. Department of Forestry, IUCN-The World Conservation Union. 112 p.

[62] International Centre for Environmental Management (2003) Lao PDR national report on protected areas and development. Available: http://www.mekong-protected-areas.org/lao_pdr/n_report.htm. Accessed 2014 Aug 3.

[63] Singleton I, Wich S, Husson S, Stephens S, Atmoko SU, et al**.**, **editors** 2004) Orangutan population and habitat viability assessment: final report. Apple Valley: IUCN/SSC Conservation Breeding Specialist Group. 235 p.

[64] Robertson Y (2002) Briefing Document on Road Network through the Leuser Ecosystem. Available: http://www-3.unipv.it/webbio/api/leuser.htm. Accessed 2014 Aug 3.

[65] van Beukering PJH, Cesar HSJ, Janssen MA (2003) Economic valuation of the Leuser National Park on Sumatra, Indonesia. Ecol Econ 44:43–62.

[66] Gray DD (2006) Ho Chi Minh Trail Vietnam, from Soldier's Road to Tourist Highway. Associated Press. Available: http://usatoday30.usatoday.com/travel/destinations/2005-07-18-ho-chi-minh_x.htm. Accessed 2014 Aug 3.

[67] Reuters (2001) Vietnam's New Highway may Cut through Reserve. Reuters Newswire. Available: http://www.iol.co.za/scitech/technology/vietnam-s-new-highway-may-cut-through-reserve-1.76017#.U95qOvmSw2I. Accessed 2014 Aug 3.

[68] Sunderlin WD (2002) Effects of crisis and political change, 1997-1999. In Colfer CJP, Resosudarmo IAP, editors. Which way forward? People, forests, and policymaking in Indonesia. Resources for the Future. Washington DC: Center for International Forestry Research (CIFOR) and Institute of Southeast Asian Studies (ISEAS). pp.246–276.

[69] Redford KH (1992) The empty forest. BioScience 42:412–422.

[70] Gaveau DLA, Sloan S, Molidena E, Yaen H, Sheil D, et al (2014) Four decades of forest persistence, clearance and logging on Borneo. PLoS ONE 9:e101654. 25029192 10.1371/journal.pone.0101654 PMC4100734

[71] Carlson KM, Curran LM, Asner GP, Pittman AM, Trigg SN, et al (2012) Carbon emissions from forest conversion by Kalimantan oil palm plantations. Nature Climate Change 3:283–287.

[72] Siegert F, Ruecker G, Hinrichs A, Hoffmann AA (2001) Increased damage from fires in logged forests during droughts caused by El Niño. Nature 414:437–440. 11719802 10.1038/35106547

[73] Vayda AP, Sahur A (1996) Bugis settlers in East Kalimantan's Kutai National Park, their past and present and some possibilities for their future. Jakarta: Center for International Forestry Research Special Publication. 54 p.

[74] Varma S, Dang NX, Thanh TV, Sukumar R (2008) The elephants *Elephas maximus* of Cat Tien National Park, Vietnam: status and conservation of a vanishing population. Oryx 42:92–99.

[75] Goosem M, Izumi Y, Turton S (2001) Efforts to restore habitat connectivity for an upland tropical rainforest fauna: a trial of underpasses below roads. Ecol Manag Restor 2:196–202.

[76] Quintero J, Roca R, Morgan AJ, Mathur A (2010) Smart Green Infrastructure in Tiger Range Countries: A Multi-level Approach. Available: http://www.globaltigerinitiative.org/download/GTI-Smart-Green-Infrastructure-Technical-Paper.pdf. Accessed 2014 Aug 3.

[77] Weston N, Goosem M, Marsh H, Cohen M, Wilson R (2011) Using canopy bridges to link habitat for arboreal mammals: successful trials in the Wet Tropics of Queensland. Australian Mammalogy 33:93–105.

[78] van der Grift EA, van der Ree R, Fahrig L, Findlay S, Houlahan J, et al (2013) Evaluating the effectiveness of road mitigation measures. Biodivers Conserv 22:425–448.

[79] Channa P, Gray T (2009) The status and habitat of yellow-cheeked crested gibbon *Nomascus gabriellae* in Phnom Prich Wildlife Sanctuary, Mondulkiri. Phnom Penh: WWF Greater Mekong-Cambodia Country Programme. 24 p.

[80] Johnson A, Vongkhamheng C, Venevongphet, Saythongdum T, Hedemark M (2006) Status of tiger, prey and human-tiger conflict in the Nam Et-Phou Louey National Protected Area, Lao PDR. In McNeely JA, McCarthy TM, Smith A, Olsvig-Whittaker L, Wikramanayake EL, editors. Conservation Biology in Asia. Kathmandu: Society for Conservation Biology Asia Section and Resources Himalaya. pp.232–235.

[81] Lynam AJ, Moore C (2013) A law enforcement action plan for reducing deforestation and forest degradation in Houaphan Province. Vientiane: Wildlife Conservation Society.

[82] Laurance WF (2001) Tropical logging and human invasions. Conserv Biol 15:4–5.

[83] Meijaard E, Sheil D, Iskandar D, Rosenbaum B, Lammertink M, et al**.** (2004) How to make wildlife conservation more compatible with production forestry: A case study from Kalimantan. Kota Kinabalu: Bornean Biodiversity and Ecosystems Conservation Programme.

[84] Linkie M, Haidir IA, Nugronho A, Dinata Y (2008) Conserving tigers *Panthera tigris* in selectively logged Sumatra forests. Biol Conserv 141:2410–2415.

[85] Meijaard E, Sheil D (2008) The persistence and conservation of Borneo's mammals in lowland rain forests managed for timber: Observations, overviews and opportunities. Ecol Res 23:21–34.

[86] Obidzinski K, Andrianto A, Wijaya C (2007) Cross-border timber trade in Indonesia: critical or overstated problem? Forest governance lessons from Kalimantan. Int For Rev 9:526–535.

[87] Asian Development Bank (2005) Greater Mekong subregion biodiversity conservation initiative: Strategic framework and technical Assessment 2005-2014. Bangkok: Asian Development Bank. 221 p.

[88] Department of Wildlife and National Parks (2008) National tiger action plan for Malaysia. Kuala Lumpur: Department of Wildlife and National Parks. 75 p.

[89] Ministry of Forestry (2007) Strategy and action plan for Sumatran Tiger (*Panthera tigris sumatrae*) conservation 2007-2017. Jakarta: Department of Forestry. 178 p.

[90] Wildlife Conservation Society (1999) Survey, assessment and conservation of the Sumatran tiger (*Panthera tigris sumatrae*) in Bukit Barisan National Park. Bogor: Wildlife Conservation Society-Indonesia.

[91] Travers H, Moore C, Johnson A (2011) Investigation of the drivers of deforestation and forest degradation in Nam Et Phou Louey National Protected Area, Lao PDR. Vientiane: Wildlife Conservation Society Lao PDR Program.

[92] Clements GR, Yap WL, Henry P (2012) Towards safer passages: The Kenyir wildlife corridor project. Malay Natural 65:56–59.

[93] Caldecott J, Miles L, editors 2005), World atlas of great apes and their conservation. Berkeley: University of California Press. 456 p.

[94] Wich SA, Meijaard E, Marshall AJ, Husson S, Ancrenaz M, et al (2008) Distribution and conservation of the orang-utan (*Pongo* spp.) on Borneo and Sumatra: how many remain? Oryx 42:329–339.

[95] Clements GR, Sayer J, Boedhihartono AK, Venter O, Lovejoy T, et al (2010) Cautious optimism over Norway-Indonesia REDD pact. Conserv Biol 24:1437–1438. 21070363 10.1111/j.1523-1739.2010.01584.x

[96] Reid J (2013) Financial mechanisms for environmental compliance in infrastructure projects. Conservation Strategy Fund, Discussion paper no. 5.

[97] Asian Development Bank (2010) HIV prevention and infrastructure: Mitigating risk in the Great Mekong subregion. Available: http://www.hivpolicy.org/Library/HPP001506.pdf. Accessed 03 Aug 2014.

[98] Marris G, Hedemark M, Johnson A, Vongkhamheng C (2002) Environmental Baseline Study of the Route 3 Upgrade through the Nam Ha protected area. Available: http://www.directoryofngos.org/project_publications/ADB%20Rt3%20baseline.pdf. Accessed 2014 Aug 3.

[99] Solichin S (2002) Fire threat from analysis in West Kutai District of East Kalimantan, Indonesia. Diploma Thesis. University of Freiburg.

[100] New Straits Times (2009) Trapped Tiger Saved, but More Patrols Needed. Available: http://bigcatrescue.blogspot.com/2009/10/tiger-rescue-points-to-urgent-need-for.html. Accessed 2014 Aug 3.

[101] New Straits Times (2011) Strong Case for Lower Belum and Temengor Conservation. Available: https://www.mns.my/article.php?aid=1552. Accessed 2014 Aug 3.

[102] TRAFFIC (2001) New Documentary Sheds Light on Poaching Crisis in Belum-Temengor. Available: http://www.traffic.org/home/2011/7/29/new-documentary-sheds-light-on-poaching-crisis-in-belum-teme.html. Accessed 2014 Aug 3.

[103] Down to Earth (2002) Aceh Pushes Leuser Road Plan. Available: http://www.downtoearth-indonesia.org/story/aceh-pushes-leuser-road-plan. Accessed 2014 Aug 3.

[104] Asian Development Bank (2008) Lao People's Democratic Republic and Socialist Republic of Vietnam: Greater Mekong subregion: east-west corridor project, Performance Evaluation Report. Available: http://www.adb.org/sites/default/files/projdocs/2008/41353-REG-TAR.pdf. Accessed 2014 Aug 3.

[105] Jepson P, Momberg F, van Noord H (2002) A review of the efficacy of the protected area system of East Kalimantan province, Indonesia. Nature Area J 22:28–42.

