# Supplementary material for: Where and How Are Roads Endangering Mammals in Southeast Asia's Forests?
Source: PLoS One. 2014 Dec 18;9(12):e115376. doi: 10.1371/journal.pone.0115376 (PMC4270763; doi:10.1371/journal.pone.0115376)
Supplement: S1 Table — Summary of 16 existing roads contributing to forest conversion of mammal habitats and hunting of endangered mammals according to 36 experts from seven countries in Southeast Asia (number of experts who responded/number of experts contacted). (DOCX) [file pone.0115376.s001.docx]

**Table S1**. Summary of 16 existing roads contributing to forest conversion of mammal habitats and hunting of endangered mammals according to 36 experts from each country (number of experts who responded/number of experts contacted) in Southeast Asia.

| **Country** | **Existing road (network)** | **Threatened endangered mammal habitats** | **Endangered mammals recorded (historically and currently) in habitats** |
| --- | --- | --- | --- |
| Cambodia (4/4) | National Highway 4 | Kirirom and Bokor NP | Asian Elephant, Banteng, Eld's Deer, Tiger, Pileated Gibbon [1], [2] |
|  | Provincial Road Network 76-141 | Eastern Plains Landscape* | Asian Elephant, Banteng, Black-shanked Douc Langur, Eld's Deer, Tiger, Yellow-cheeked Crested Gibbon [3] |
|  | National Road 48 | Cardamom Mountains^ | Asian Elephant, Dhole, Pileated Gibbon, Tiger [4] |
| Indonesia |  |  |  |
| *Kalimantan (5/5)* | Bontang-Sangata Road | Kutai NP | Banteng, Bornean Orangutan, Bornean Gibbon [5], [6], [7] |
|  | Balikpapan-Samarinda Road | Bukit Soeharto RF | Bornean Gibbon, Sunda Otter Civet [8], [9] |
|  | Logging road networks | Priority sites for Orangutan conservation# | Banteng, Bornean Orangutan [10], [5] |
| *Sumatra (7/8)* | Sanggi-Bengkunat/Krui Liwa Roads | Bukit Barisan Selatan NP | Agile Gibbon, Asian Elephant, Asian Tapir, Siamang, Sumatran Rhino, Tiger [11] |
|  | Blangkejeren-Kutacane Road | Gunung Leuser NP | Asian Elephant, Sumatran Orangutan, Sumatran Rhino, Tiger [12] |
|  | Logging road networks | Tiger conservation landscapes† | Asian Elephant, Sumatran Orangutan, Tiger [13], [14] |
| Lao PDR (3/3) | Route 9 | Phou Xang He and Dong Phou Vieng NBCAs | Asian Elephant, Douc Langur, Giant Muntjac, Tiger Cleetus [15] |
|  | Route Network 12-1E-8 | Nakai-Nam Theun NBCA | Asian Elephant, Dhole, Douc Langur, Giant Muntjac, Yellow-cheeked Crested Gibbon, Saola, Tiger [16], [17] |
|  | Route Network 17A-3 | Nam Ha NBCA | Asian Elephant, Black-crested Gibbon, Dhole, Tiger [18], [19] |
| Malaysia |  |  |  |
| *East (5/7)* | Kalabakan-Sapulut Road | FRs in Tawau and Pensiangan Districts | Asian Elephant, Sumatran Rhino [20], [21] |
|  | Logging road networks | FRs, Kelabit highlands | Banteng, Bornean Gibbon, Sumatran Rhino [22] |
|  | Access roads for dams | Murum, Danum and Pileran Valleys | Bornean Gibbon [23] |
| *Peninsular (7/9)* | Federal Route 4 | Royal Belum State Park, Temengor FR | Asian Elephant, Asian Tapir, Siamang, Sunda Pangolin, Tiger, White-handed Gibbon [24] |
|  | Federal Route 8 | Taman Negara NP, Titiwangsa Main Range | Asian Elephant, Asian Tapir, Dhole, Siamang, Sunda Pangolin, Tiger, White-handed Gibbon [25] |
|  | State Route T156 | Tembat, Petuang and Hulu Telemong FRs | Asian Elephant, Asian Tapir, Dhole, Sunda Pangolin, Tiger, White-handed Gibbon [26] |
| Myanmar (1/3) | Wildlife trade route network | All mammal habitats in Myanmar | See Results |
|  | Roads in E, W and NW sector | Alaungdaw Kathapa NP | Asian Elephant, Banteng, Dhole, Tiger [27], [28] |
|  | Ledo road | Hukaung Valley WS | Tiger [29] |
| Vietnam (3/3) | Ho Chi Minh Highway | Protected areas§ | Asian Elephant, Delacour's Langur, Northern White-cheeked Gibbon, Red-shanked Douc, Saola,[30], [31] |
|  | Roads in banteng habitats | Ea So, Yok Don and Krong Trai NR, Vinh Cuu NP | Banteng [32] |
|  | Roads in | Cat Tien NP | Asian Elephant, Javan Rhino (hunted to extinction during time of writing) [33], [34] |
|  |  |  |  |

* Mondulkiri PF, Seima BCA, Lumphat, Snuol, Phnum Prech and Phnum Namlier WS

^ Phnum Samkos and Phnum Aural WS, Central Cardamom PF

# Gunung Palung, Danau Sentarum/Bentung Kerihun, Tanjung Puting, Belantikan, Gunung Gajah/Berau/Kelai, Sebangau

† Kerinci Seblat NP, Tesso Nilo and Bukit Tigapuluh landscapes, Bukit Rambang Baling, Kuala Kampar-Kerumutan, Rimbo Panti-Batang Gadu, proHUsed Senepis-Buluhala Tiger National Park

§ Cuc Phuong and Phong Nha-Ke Bang NP, Vu Quang NR

NOTE: BCA = Biodiversity Conservation Area; FR = Forest Reserve; PA = Protected Area; PF = Protection Forest; NBCA = National Biodiversity Conservation Area; NP = National Park; NS = Nature Reserve; RF = Recreation Forest; WS = Wildlife Sanctuary

**References**

1. Protected Areas Development (2004) Field study: Cambodia, Bokor, Kirirom, Kep and Ream National Park, Protected Areas and Development in the Lower Mekong River Region. Available: http://www.mekong-protected-areas.org/cambodia/docs/cambodia_field.pdf. Accessed 03 Aug 2014.
2. Http 1: Kirirom National Park. Available: http://en.wikipedia.org/wiki/Kirirom_National_Park. Accessed 03 Aug 2014.
3. Walston J, Davidson P, Men Soriyun NY (2001) A wildlife survey of Southern Mondulkiri Province, Cambodia. Phnom Penh: Wildlife Conservation Society (Cambodia Programme). 80 p.
4. Daltry JC, Momberg, F (2000) Biological survey of the Cardamom mountains, southwestern Cambodia. Cambridge: Fauna and Flora International, Government of Cambodia, Ministry of Environment and Wildlife Protection Office. 20 p.
5. Wich SA, Meijaard E, Marshall AJ, Husson S, Ancrenaz M, et al. (2008) Distribution and conservation of the orang-utan (*Pongo* spp.) on Borneo and Sumatra: how many remain? Oryx 42: 329-339.
6. Setiawan A, Nugroho TS, Djuwantoko, Pudyatmoko S (2009) A survey of Miller’s Grizzled Surili*, Presbytis hosei canicurus*, in East Kalimantan, Indonesia. Primate Conserv 24: 139-143.
7. MONGABAY.COM (2009) Orangutan Population in Borneo Park Plunges 90% in 5 years. MONGABAY.COM. Available: http://news.mongabay.com/2009/0517-Orangutans_kutai.html. Accessed 03 Aug 2014.
8. Yasuma S (1994) An invitation to the mammals of East Kalimantan. Jakarta: Pusrehut Special Publication no. 3, Tropical Rainforest Project, Japan International Cooperation Agency, Directorate General of Higher Education and Ministry of Education and Culture. 384 p.
9. Oka T, Uskander E, Ghozali DI (2000) Effects of forest fragmentation on the behaviour of Bornean gibbons. In: Guhardja E, Fatawi M, Sutisna M, Mori T, Ohta S, editors. Rainforest ecosystems of East Kalimantan: El Nino, drought, fire and human impacts. Tokyo: Ecological studies 140, Springer-Verlag. pp. 229-238.
10. Orangutan Conservation Services Program (2007) OCSP threat analysis and site selection for Kalimantan and Sumatra. Available: http://pdf.usaid.gov/pdf_docs/Pnadl978.pdf. Accessed 03 Aug 2014.
11. O'Brien T, Kinnaird MF (1996) Birds and mammals of the Bukit Barisan Selatan National Park, Sumatra, Indonesia Oryx: 30: 207-217.
12. Singleton I, Wich S, Husson S, Stephens S, Atmoko SU et al., editors (2004) Orangutan population and habitat viability assessment: final report. Apple Valley: IUCN/SSC Conservation Breeding Specialist Group. 235 p.
13. Dinerstein E, Loucks C, Heydlauff A, Wikramanayake E, Bryja, G et al. (2006) Setting priorities for the conservation and recovery of wild tigers: 2005-2015: A user's guide. Washington DC: World Wildlife Fund, Wildlife Conservation Society, Smithsonian, National Fish and Wildlife Foundation-Save the Tiger Fund. 50 p.
14. Eyes on the Forest (2008) Asia Pulp and Paper threatens Senepis forest, Sumatran tiger habitat, and global climate. Riau: Eyes on the Forest 15 p..
15. Cleetus R (2005) Lao PDR: Using strategic environmental vulnerabilities assessment (SEVA) for evaluating threats to forests. Washington DC: WWF-Macroeconomics Program Office. 7 p.
16. Timmins RJ, Evans TD (1996) A wildlife and habitat survey of the Nakai-Nam Theun National Biodiversity Conservation Area, Khammouane and Bolikhamsai Provinces, Laos. Vientiane: Department of Forestry, Ministry of Agriculture and Forestry, The Wildlife Conservation Society. 59 p.
17. Timmins RJ, Duckworth JW (2004) Status and conservation of Douc Langurs (*Pygathrix nemaeus*) in Laos. Int J Primatol 20: 469-489.
18. Tizard R, Davidson P, Kamkhoun, Salivong K (1997) A wildlife and habitat survey of Nam Ha and Nam Kong Protected Areas, Luang Namtha Province, Lao PDR. Vientiane: Department of Resource Conservation, Wildlife Conservation Society Cooperative Program, Department of Forestry.
19. Johnson A, Singh S, Duangdala M, Hedemark M (2005) The western black crested gibbon *Nomascus concolor* in Laos, new records and conservation status. Oryx 39: 311-317.
20. Unet R (2009) Concern over Sapulut-Kalabakan Highway. Available: http://sabahmajujaya.blogspot.com/2009/03/concern-over-sapulut-kalabakan-highway.html. Accessed 03 Aug 2014.
21. Ambu LN, Andua PM, Nathan S, Tuuga A, Jensen SM et al. Asian Elephant Action Plan Sabah (Malaysia). Kota Kinabalu: Sabah Wildlife Department.
22. Abdullah MT, Lakim M, Abdul Rahman MA (1999) Notes on large mammals of Bario, Sarawak. In: Ismail G, Laily DD, editors. A scientific journey through Borneo, Bario, the Kelabit highlands of Sarawak. Kuching: Pelanduk Publications. pp. 221-222.
23. Then S (02 Nov 2009) Murum Dam: More than 100 Wildlife Species at Risk. The Star. Available: http://www.thestar.com.my/story.aspx?file=%2f2009%2f11%2f2%2fnation%2f20091102152540&sec=nation. Accessed 03 Aug 2014.
24. Rayan DM, Lau CF, Goh SS, Mohamad S, Wong CTC, Siwan ES, Hamirul M, Mohamed A (2012) Management recommendations on ecological linkages: findings from a study on large mammal habitat use within the Belum-Temengor corridor. Petaling Jaya: WWF-Malaysia. 35 p.
25. Kawanishi K, Sunquist ME (2004) Conservation status of tigers in a primary rainforest of Peninsular Malaysia. Biol Conserv 120: 329-344.
26. Clements GR (2013) The environmental and social impacts of roads in Southeast Asia. Ph.D. Thesis, James Cook University. Available: http://researchonline.jcu.edu.au/31888/, Accessed 03 Aug 2014.
27. Wildlife Conservation Society (2002) Development of an Action Plan; Myanmar Tiger Conservation - II. Yangon. Available: http://www.ibiblio.org/obl/docs2/MyanmarReport1.pdf. Accessed 03 Aug 2014.
28. Lynam AJ, Rabinowitz A, Myint T, Maung M, Latt KT et al. (2009) Estimating abundance with sparse data: tigers in northern Myanmar. Popul Ecol 51: 115-121.
29. Rabinowitz A (2004) A question of balance. Natl Geogr 205: 98-117.
30. Eve R, Madhavan S, Dzung VV (2000) Spatial planning for nature conservation in Vu Quang Nature Reserve: a landscape ecology approach. Hanoi: World Wide Fund for Nature-Indochina Program. 158 p.
31. Reuters (29 Oct 2001) Vietnam’s New Highway may Cut through Reserve. Reuters Newswire. Available: http://www.iol.co.za/scitech/technology/vietnam-s-new-highway-may-cut-through-reserve-1.76017#.U95qOvmSw2I. Accessed 03 Aug 2014.
32. Pedrono M, Tuan HA, Chouteau P, Vallejo F (2009) Status and distribution of the endangered banteng (*Bos javinicus birmanicus*) in Vietnam: a conservation tragedy. Oryx 43: 618-625.
33. Polet G, Ling S (2004) Protecting mammal diversity: opportunities and constraints for pragmatic conservation management in Cat Tien National Park, Vietnam. Oryx 38: 186-196.
34. Brook SM, van Coeverden de Groot P, Scott C, Boag P, Long B et al. (2012) **Integrated and novel survey methods for rhinoceros populations confirm the extinction of *Rhinoceros sondaicus annamiticus* from Vietnam**. Biol Conserv 155: 59-67.
