# Supplementary material for: Where and How Are Roads Endangering Mammals in Southeast Asia's Forests?
Source: PLoS One. 2014 Dec 18;9(12):e115376. doi: 10.1371/journal.pone.0115376 (PMC4270763; doi:10.1371/journal.pone.0115376)
Supplement: S2 Table — Summary of 8 planned road construction or improvement projects that can potentially contribute to forest conversion of mammal habitats and hunting of endangered mammals according to experts from five Southeast Asian countries. (DOCX) [file pone.0115376.s002.docx]

**Table S2**. Summary of 8 planned road construction or improvement projects that can potentially contribute to forest conversion of mammal habitats and hunting of endangered mammals according to experts from five Southeast Asian countries.

| **Country** | **Planned road construction or upgrading project** | **Threatened endangered mammal habitats** | **Endangered mammals in habitats** |
| --- | --- | --- | --- |
| Cambodia | Expansion of National Road 48 | Phnum Samkos and Phnum Aural Wildlife Sanctuaries, Central Cardamom Protection Forest | Asian Elephant, Dhole, Pileated Gibbon, Tiger [1] |
|  | Expansion of logging road to link National Road 48 and Samkos | Phnum Samkos and Phnum Aural Wildlife Sanctuaries, Central Cardamom Protection Forest | Asian Elephant, Dhole, Pileated Gibbon, Tiger [2] |
| Indonesia |  |  |  |
| *Kalimantan* | Kalimantan Border Oil Palm Mega-Project | Bentung Kerihun National Park | Bornean Orangutan [3] |
|  | Balang Island Bridge Project | Sungai Wain Protection Forest | Bornean Gibbon, Bornean Orangutan, Bay Cat [4] |
| *Sumatra* | Ladia Galaska Scheme | Gunung Leuser National Park | Sumatran Orangutan [5] |
| Lao PDR | Upgrading of Route 18 | Xe Pian National Biodiversity Conservation Area | NA |
| Malaysia |  |  |  |
| *Peninsular* | Kuala Lumpur Outer Ring Road | Selangor State Park | Asian Tapir [6] |
| Myanmar | Upgrading of Dawei-Myeik-Kyawthaung Highway | Contributes to Thailand-Myanmar-China smuggling route | Mammals targeted by wildlife trade |
| Vietnam | Road in northern section of Mondulkiri Protection Forest | Mondulkiri Protection Forest | [7] |

**References**

1. Asian Development Bank (2005) Greater Mekong subregion biodiversity conservation initiative: Strategic framework and technical Assessment 2005-2014. Bangkok: Asian Development Bank. 221 p.
2. Sovan N (11 Aug 2008) Dams Threaten Siamese Crocs. The Phnom Penh Post. Available: http://www.phnompenhpost.com/national/dams-threaten-siamese-crocs. Accessed 03 Aug 2014.
3. Wakker E (2006) The Kalimantan border oil palm mega-project. Amsterdam: Milieudefensie – Friends of the Earth Netherlands and Swedish Society for Nature Conservation. 50 p.
4. Hance J (2010) Bridge development in Kalimantan threatens rainforest, mangroves, and coral reef. MONGABAY.COM. Available: http://news.mongabay.com/2010/0103-hance_pulau.html. Accessed 03 Aug 2014.
5. Gaveau DLA, Wich S, Epting J, Juhn D, Kanninen M, et al. (2009) The future of forests and Orangutans (*Pongo abelii*) in Sumatra: predicting impacts of oil palm plantations, road construction, and mechanisms for reducing carbon emissions from deforestation. Environ Res Lett 4: 034013.
6. Http 1 (2013) Protect Klang Gates Quartz Ridge and Selangor State Park from the Proposed KL Outer Ring Road. Available: http://www.petitiononline.com/KLORR/petition.html. Accessed 03 Aug 2014.
7. International Organization for Migration (2009) Mapping vulnerability to natural hazards in Mondulkiri. Phnom Penh: International Organization for Migration Mission. 109 p.
