# Supplementary material for: Where and How Are Roads Endangering Mammals in Southeast Asia's Forests?
Source: PLoS One. 2014 Dec 18;9(12):e115376. doi: 10.1371/journal.pone.0115376 (PMC4270763; doi:10.1371/journal.pone.0115376)
Supplement: S3 Table — Logistic regression models examining the effect of four site covariates on the endangered Asian Tapir ( Tapirus indicus ) habitat use ( ψ ), and three sampling covariates affecting its detection probability ( p ), based on camera-trap data from forests along State Road 156, a road identified by one of the experts in Peninsular Malaysia. (DOCX) [file pone.0115376.s003.docx]

**Table S3.** Logistic regression models examining the effect of four site covariates on the endangered Asian Tapir (*Tapirus indicus*) habitat use (*ψ*), and three sampling covariates affecting its detection probability (*p*), based on camera-trap data from forests along State Road 156, a road identified by one of the experts in Peninsular Malaysia.

| **Candidate models** | **AICc** | **∆AICc** | **wAICc** | **k** | **DE** | **%DE** | ***__*** | **ER** |
| --- | --- | --- | --- | --- | --- | --- | --- | --- |
|  |  |  |  |  |  |  |  |  |
| *ψ*(resv),*p*(trap+rain) | 822.17 | 0.00 | 0.25 | 5 | 811.78 | 0.45 | 0.89 | 1.36 |
| *ψ*(resv+plan),*p*(trap+rain) | 822.79 | 0.62 | 0.18 | 6 | 810.23 | 0.64 | 0.93 |  |
| *ψ*(.),*p*(trap+rain) | 823.73 | 1.56 | 0.11 | 4 | 815.47 | 0.00 | 0.92 |  |
| *ψ*(resv+road)*,p*(trap+rain) | 823.81 | 1.64 | 0.11 | 6 | 811.25 | 0.52 | 0.86 |  |

Note: Only candidate models with ∆AICc < 2 are shown. The top-ranked model was used to generate habitat-use-intensity maps. Site covariates included in each model are: 1) road = distance to edge of State Route T156; 2) plan = distance to nearest plantation edge; and 3) resv = distance to reservoir edge. AIC_c_ = Akaike’s Information Criterion corrected for small sample size; ∆AIC_c_ = difference in AIC_c_ for each model from the most parsimonious model; wAIC_c_ = AIC_c_ weight, k = number of parameters; DE = deviance; % DE = % deviance explained in the response variable by the model under consideration;__ = overdispersion factor. Sampling covariates included in each model are: 1) trap = no. of trap nights that cameras were operational during each sampling occasion; and 2) rain = daily rainfall.
