# Supplementary material for: Where and How Are Roads Endangering Mammals in Southeast Asia's Forests?
Source: PLoS One. 2014 Dec 18;9(12):e115376. doi: 10.1371/journal.pone.0115376 (PMC4270763; doi:10.1371/journal.pone.0115376)
Supplement: S4 Table — Confusion matrix used in accuracy analysis of 2010 classified image from Snuol Wildlife Reserve, Cambodia. (DOCX) [file pone.0115376.s004.docx]

**Table S4*.*** Confusion matrix used in accuracy analysis of 2010 classified image from Snuol Wildlife Reserve, Cambodia.

|  |  | **Ground Points** | | | | |  |  |
| --- | --- | --- | --- | --- | --- | --- | --- | --- |
|  |  | Bare or built-up | Mosaic | Mature Forest | Others | Water | **Subtotal (Classified Pixel)** | **User's Accuracy (%)** |
| **Classified Pixel** | Bare or built-up | 118 | 18 | 0 | 3 | 0 | 139 | 84.9 |
|  | Mosaic | 5 | 137 | 20 | 2 | 0 | 164 | 83.5 |
|  | Mature Forest | 0 | 15 | 155 | 0 | 0 | 170 | 91.2 |
|  | Others | 6 | 5 | 0 | 9 | 1 | 21 | 42.9 |
|  | Water | 0 | 0 | 0 | 1 | 5 | 6 | 83.3 |
|  | **Subtotal (Ground Points)** | 129 | 175 | 175 | 15 | 6 | 500 |  |
|  | **Producer's Accuracy (%)** | 91.5 | 78.3 | 88.6 | 60.0 | 83.3 |  |  |
|  | **Overall Accuracy (%)** | 84.8 |  |  |  |  |  |  |

Note: Classification accuracy for the image was estimated on a per pixel basis using reference datasets of 500 randomly generated points. The reference datasets consisted of the original Landsat 5 image and a Landsat 7 image obtained from a similar temporal period. These reference datasets were used because there was limited overlap in historical imagery available in Google Earth. As we could not obtain high spatial resolution images for ~1990 and ~2000, we could only assess the accuracy of the 2010 image. However, we expect the accuracy of classified images for 1990 and 2000 to be comparable to that of the 2010 image because it was produced with the same data and methods. Overall accuracy was relatively high at 85% and the producer’s accuracy was also generally high for all land-cover classes, ranging from 60-91%.
