# Supplementary material for: Where and How Are Roads Endangering Mammals in Southeast Asia's Forests?
Source: PLoS One. 2014 Dec 18;9(12):e115376. doi: 10.1371/journal.pone.0115376 (PMC4270763; doi:10.1371/journal.pone.0115376)
Supplement: S5 Table — Summary statistics for transition of land categories to mosaic of secondary forests from 1990–2001 and 2001–2009, and transition of categories to bare or built-up areas in 1990–2001and 2001–2009 in Snuol Wildlife Reserve, Cambodia. Each row respectively gives: a) land category name, b) area of transition in terms of cell counts, c) intensity of transition per gross gain, d) uniform distribution of transitions across the area possible for that change, given the empirical gross gain for mosaic or bare or built-up areas, e) hypothesized uniform annual transition, f) annual number of pixels of hypothesized error, g) commission or omission intensity in t map and h) hypothesized error as percent of t map. (DOCX) [file pone.0115376.s005.docx]

|  | 1990 to 2001 | | | | | | | |
| --- | --- | --- | --- | --- | --- | --- | --- | --- |
| transitions TO Mosaic | | | | | | | | |
| FROM | Observed | Intensity of | Uniform | Hypothesized | Annual # of pixels | Commission | Ommission | Error as % |
| Category | transition | transition | distribution | annual transition | of hypothesized error | intensity | intensity | of map1990 |
| Primary forest | 835 | 2.06 | 2.40 | 1020 | 185 | 0.00 | 14.80 | 3.31 |
| Bare or Built-up | 150 | 5.90 | 2.40 | 29 | 121 | 11.38 | 0.00 | 3.31 |
| Other | 78 | 5.91 | 2.40 | 15 | 63 | 5.91 | 0.00 | 3.31 |
| Water | 2 | 3.54 | 2.40 | 1 | 1 | 0.09 | 0.00 | 3.31 |
|  | 2001 to 2009 | | | | | | | |
| transitions TO Mosaic | | | | | | | | |
| FROM | Observed | Intensity of | Uniform | Hypothesized | Annual # of pixels | Commission | Ommission | Error as % |
| Category | transition | transition | distribution | annual transition | of hypothesized error | intensity | intensity | of map1990 |
| Primary forest | 1268 | 3.31 | 3.20 | 1214 | 54 | 4.21 | 0.00 | 0.71 |
| Bare or Built-up | 17 | 1.06 | 3.20 | 61 | 45 | 0.00 | 3.34 | 0.71 |
| Other | 8 | 1.71 | 3.20 | 18 | 9 | 0.00 | 0.73 | 0.71 |
| Water | 0 | 0.00 | 3.20 | 0 | 0 | 0.00 | 0.01 | 0.71 |

**Table S5.** Summary statistics for transition of land categories to mosaic of secondary forests from 1990-2001 and 2001-2009, and transition of categories to bare or built-up areas in 1990-2001and 2001-2009 in Snuol Wildlife Reserve, Cambodia. Each row respectively gives: a) land category name, b) area of transition in terms of cell counts, c) intensity of transition per gross gain, d) uniform distribution of transitions across the area possible for that change, given the empirical gross gain for mosaic or bare or built-up areas, e) hypothesized uniform annual transition, f) annual number of pixels of hypothesized error, g) commission or omission intensity in t map and h) hypothesized error as percent of t map.

|  | 1990 to 2001 | | | | | | | |
| --- | --- | --- | --- | --- | --- | --- | --- | --- |
| transitions TO Bare or Built-up | | | | | | | | |
| FROM | Observed | Intensity of | Uniform | Hypothesized | Annual # of pixels | Commission | Ommission | Error as % |
| Category | transition | transition | distribution | annual transition | of hypothesized error | intensity | intensity | of map1990 |
| Primary forest | 27 | 0.07 | 0.12 | 49 | 22 | 0.00 | 23.34 | 0.39 |
| Mosaic | 24 | 0.14 | 0.12 | 21 | 3 | 4.43 | 0.00 | 0.39 |
| Other | 19 | 1.41 | 0.12 | 1 | 17 | 24.17 | 0.00 | 0.39 |
| Water | 1 | 2.31 | 0.12 | 0 | 1 | 1.84 | 0.00 | 0.39 |
|  | 2001 to 2009 | | | | | | | |
| transitions TO Bare or Built-up | | | | | | | | |
| FROM | Observed | Intensity of | Uniform | Hypothesized | Annual # of pixels | Commission | Ommission | Error as % |
| Category | transition | transition | distribution | annual transition | of hypothesized error | intensity | intensity | of map1990 |
| Primary forest | 771 | 2.01 | 2.20 | 859 | 89 | 0.00 | 6.29 | 1.19 |
| Mosaic | 543 | 2.56 | 2.20 | 451 | 92 | 6.95 | 0.00 | 1.19 |
| Other | 8 | 1.69 | 2.20 | 11 | 3 | 0.00 | 0.22 | 1.19 |
| Water | 0 | 0.00 | 2.20 | 0 | 0 | 0.00 | 0.01 | 1.19 |

Note: Coloured cells present the observed intensity in terms of the percent of the category at t map of each interval, the area of transition within the interval, and the omission or commission errors in t map: 1) green cells: indicates that the category targets that transition; 2) red cells: indicates that the category avoids that transition; 3) pink: omission error; 4) dark gray: commission error; and 5) black: transition from category to mosaic or bare or built-up areas.
