# Supplementary material for: Where and How Are Roads Endangering Mammals in Southeast Asia's Forests?
Source: PLoS One. 2014 Dec 18;9(12):e115376. doi: 10.1371/journal.pone.0115376 (PMC4270763; doi:10.1371/journal.pone.0115376)
Supplement: S1 Method — Hierarchically-nested combinations of relevant keywords and wildcards used to search for road-specific biodiversity studies in Southeast Asia in the BIOSIS Previews database between 1985 and 2011. (DOCX) [file pone.0115376.s008.docx]

**Method S1.** Hierarchically-nested combinations of relevant keywords and wildcards used to search for road-specific biodiversity studies in Southeast Asia in the BIOSIS Previews^®^ database between 1985 and 2011.

TI=(Biodiversity OR Conserv* OR Deforest* OR Diversity OR Ecolog* OR Extinction* OR Fauna* OR Flora* OR Forest* OR Fragment* OR Habitat OR Rainforest OR Species OR Wildlife) AND TI=(Road* OR Highway* OR Expressway OR Overpass* OR Over-pass OR Underpass* OR Under-pass* OR Viaduct OR Culvert OR Traffic OR Vehic* OR Road kill OR Roadkill) AND TI= (Southeast Asia OR South East Asia OR SE Asia OR Borneo OR Brunei OR Indo* OR Malay* OR Philippine* OR Indo-China OR Indochin* OR Irian Jaya* Java* OR Kalimantan OR Cambodia* OR Lao* OR Burm* OR Myanmar OR Peninsular Malaysia OR Sabah OR Sarawak OR Singapore* OR Sumatra* OR Thailand OR Vietnam* OR Viet Nam OR East Timor OR Timor Leste OR Timor-Leste).
