# Supplementary material for: Where and How Are Roads Endangering Mammals in Southeast Asia's Forests?
Source: PLoS One. 2014 Dec 18;9(12):e115376. doi: 10.1371/journal.pone.0115376 (PMC4270763; doi:10.1371/journal.pone.0115376)
Supplement: S2 Method — Method used to generate habitat-use-intensity maps for the Asian Tapir ( Tapirus indicus ) from forests on either side of State Road 156, a road identified by one of the experts in Peninsular Malaysia. (DOCX) [file pone.0115376.s009.docx]

**Method S2.** Method used to generate habitat-use-intensity maps for the Asian Tapir (*Tapirus indicus*) from forests on either side of State Road 156, a road identified by one of the experts in Peninsular Malaysia.

Using a likelihood-based approach [1], [2] habitat use ($\hat{\psi}$) by this species was estimated from 158 1-km^2^ cells. Detection histories (H) were constructed over four temporal sampling occasions (15 trap nights each) to calculate detection probabilities (*p*) accounting for imperfect detection. For example, a detection history for cell *i* (H*_i_*) of ‘1001’ from four sampling occasions represents detection on the 1^st^ and 4^th^ occasions, and non-detection on the 2^nd^ and 3^rd^ occasions. The probability of recording history H*_i_* would be,

Pr (H*_i_* = 1001) = ψ*_i_* [*p_i_*_1_ (1 – *p_i_*_2_) (1 – *p_i_*_3_) *p_i_*_4_]

where ψ*_i_* is the probability that cell *i* is occupied and *p_i j_* is the probability of detecting the species at cell *i* during sampling duration *j* (= 1, 2, 3 and 4), conditional upon the species being present.

To explicitly account for variation in detection probability (*p*), two sampling covariates were included in models: (1) number of trap nights that cameras were operational during each sampling occasion; and (2) daily rainfall recorded from closest official weather station. Four site covariates that could affect Asian Tapir habitat use were also included: 1) distance to State Road 156; 2) distance to nearest plantation; 3) distance to a nearby reservoir; and 4) forest cover type as a proxy of logging intensity (1 - relatively intact lowland forest, 2 – disturbed lowland forest from a 2010 land cover layer derived from MODIS 250-m resolution satellite images; [3]). Site covariates were measured at the centroid of each 1-km^2^ cell. After testing for collinearity among continuous and categorical covariates using the *hetcor* function implemented in the polycor library in R statistical environment 3.0.0 [4], we retained covariates with coefficients <|0.5| for model construction. All continuous covariates were normalized to z-scores prior to modeling.

To account for imperfect detection, we adopted a two-step process under the single-species, single-season occupancy framework in PRESENCE v5.3 software [5]. First, to model detection probability (*p*), this parameter was assumed constant or allowed to vary with individual sampling covariates or with both combined, while all site covariates were included in each model [6]. The top-ranked model for detection probability was then used to examine the influence of site covariates on habitat use (*ψ*). This parameter was assumed constant or allowed to vary with individual or additively combined site covariates. Models were ranked using Akaike’s Information Criterion (AIC) corrected for small sample size and evaluated for goodness-of-fit against 999 simulated bootstrap datasets [7]. The top-ranked model was used to map habitat-use intensities at 1 km^2^ resolution and four levels of habitat-use intensities were defined with the Spatial Join function in ArcGIS v10 based on natural breaks (ESRI, Redlands).

**References**

1. MacKenzie DI, Nichols JD, Lachman GB, Droege S, Royle JA et al. (2002) Estimating site occupancy rates when detection probabilities are less than one. Ecology 83: 2248-2255.
2. MacKenzie DI, Nichols JD, Sutton N, Kawanishi K, Bailey LL (2005) Improving inferences in population studies of rare species that are detected imperfectly. Ecology 86: 1101-1113.
3. Miettinen J, Shi C, Tan WJ, Liew SC (2012) 2010 land cover map of insular Southeast Asia in 250-m spatial resolution. Remote Sens Lett 3: 11-20.
4. R Development Core Team (2013) R: A language and environment for statistical computing. Vienna: R Foundation for Statistical Computing.
5. Hines JE (2006) Presence V5.3 - software to estimate patch occupancy and related parameters. Laurel: United States Geological Service-Patuxent Wildlife Reseach Center. Available: http://www.mbr-pwrc.gov/software/presence.html. Accessed 05 Aug 2014
6. MacKenzie DI (2006) Modeling the probability of resource use: The effect of, and dealing with, detecting a species imperfectly. J Wildlife Manag 70: 367-374.
7. MacKenzie DI, Bailey LI, Nichols JD (2004) Investigating species co-occurrence patterns when species are detected imperfectly. J Anim Ecol 73: 546-555.
