# Supplementary material for: Where and How Are Roads Endangering Mammals in Southeast Asia's Forests?
Source: PLoS One. 2014 Dec 18;9(12):e115376. doi: 10.1371/journal.pone.0115376 (PMC4270763; doi:10.1371/journal.pone.0115376)
Supplement: S3 Method — Method for intensity analysis used to investigate whether forest conversion intensified following road construction in Snuol Wildlife Reserve, Cambodia. (DOCX) [file pone.0115376.s010.docx]

**Method S3.** Method for intensity analysis used to investigate whether forest conversion intensified following road construction in Snuol Wildlife Reserve, Cambodia.

Cross-tabulation matrices analyzed the intensity of land category change for two time intervals (1990-2001 [$Y_{t}$] and 2001-2009 [$Y_{t+1}]$). First, we analyzed variation in the annual rate of change during each time interval ($Y_{t},Y_{t+1})$, by comparing observed rates (*S_t_*) to a uniform rate (*U*) that would exist if annual changes were distributed uniformly across the entire time duration:

$S_{t}=\frac{area of change during interval [Y_{t},Y_{t+1}]/area of Snuol}{duration of interval [Y_{t},Y_{t+1}]} \times100\%$

$U=\frac{area of change during all intervals/area of Snuol}{duration of all intervals} \times100\%$

Second, we determined whether land-cover categories were relatively dormant or active during category conversions by comparing the observed intensities of gross gains (*G_tj_*) and losses (*L_ti_*) for each category with a uniform intensity ($S_{t}$) of annual change that would exist if the change during each interval was distributed uniformly across the entire spatial extent.

$G_{tj}=\frac{area of gross gain of category j\mathrm{during}\left[ Y_{t},Y_{t+1} \right]/{duration of \left[ Y_{t},Y_{t+1} \right]}}{area of category j at time Y_{t+1}} \times100\%$

$L_{ti}=\frac{area of gross loss of category i\mathrm{during}\left[ Y_{t},Y_{t+1} \right]/{duration of \left[ Y_{t},Y_{t+1} \right]}}{area of category i at time Y_{t+1}} \times100\%$

Third, we determined whether primary forests or mosaics (secondary forests/regrowth) were more likely to transition to bare or built-up areas by comparing the observed intensity of each transition ($R_{tin})$ with a uniform intensity ($W_{tn})$that would exist if the change during each interval were distributed uniformly among these land-cover categories.

$R_{tin}=\frac{area of transition from i\mathrm{to} n\mathrm{during}\left[ Y_{t},Y_{t+1} \right]/{duration of \left[ Y_{t},Y_{t+1} \right]}}{area of category i at time Y_{t+1}} \times100\%$

$W_{tn}=\frac{area of gross gain of category n\mathrm{during}\left[ Y_{t},Y_{t+1} \right]/{duration of \left[ Y_{t},Y_{t+1} \right]}}{area of category i at time Y_{t+1}} \times100\%$
