# Supplementary material for: Where and How Are Roads Endangering Mammals in Southeast Asia's Forests?
Source: PLoS One. 2014 Dec 18;9(12):e115376. doi: 10.1371/journal.pone.0115376 (PMC4270763; doi:10.1371/journal.pone.0115376)
Supplement: S1 Box — Case study of real-world challenges faced during the implementation of recommended road impact mitigation measures in Kalimantan, Indonesia. (DOCX) [file pone.0115376.s011.docx]

**Box S1.** Case study of real-world challenges faced during the implementation of recommended road impact mitigation measures in Kalimantan, Indonesia.

While our recommended best management practices have been used by various conservation and environmentalist groups, results have been mixed and successes relatively limited. The real-world example of a controversial road project in East Kalimantan, Indonesia, demonstrates how four of our best management practices have encountered obstacles during their implementation. The planned connecting road to the Pulau Balang bridge is likely to sever habitat connectivity, cause the destruction of fish breeding areas and coral reefs and promote legal and illegal settlements, logging, poaching and forest fires. One of the authors (Stanislav Lhota) has been actively involved in the campaign and negotiations opposing the Pulau Balang road since 2006. While previous local governments and NGOs have opposed this road in favor of a more cost-effective alternative route, influential individuals in the provincial (East Kalimantan Province) and current local governments are mostly in favor of this project because they allegedly stand to gain benefits from land speculation [1].


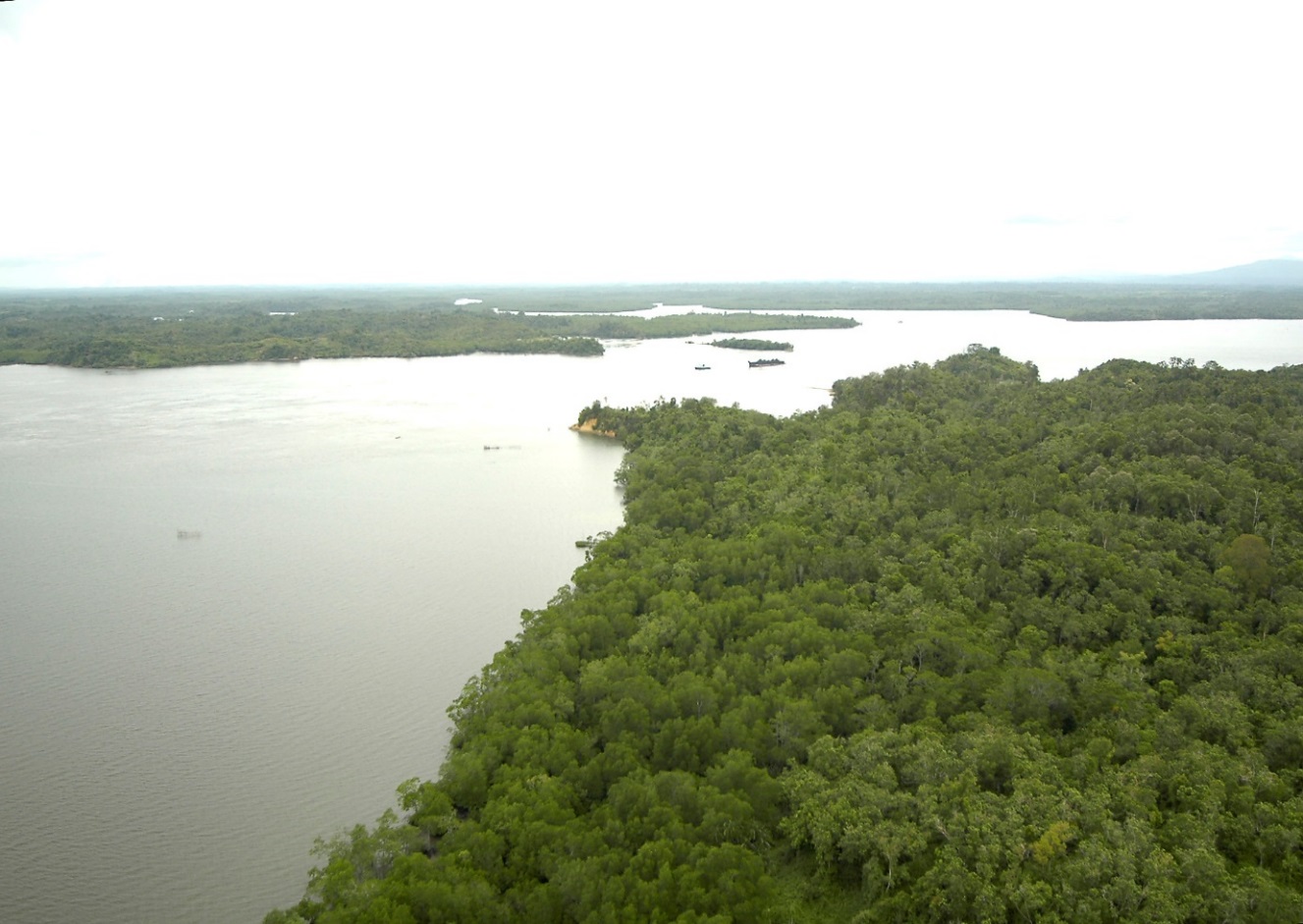


*Increase engagement with road developers in conservation planning*. At the time of the Pulau Balang road planning, two important multi-stakeholder management bodies were established with the vision of sustainable management for the area. Both pushed their own conservation plans. The Sungai Wain Protection Forest Management Body (responsible for the area east of the proposed road) stressed the threat of the Pulau Balang road in its strategic plan [2] as one of the major issues and proposed an alternative road as an economically more feasible solution, which would greatly minimize environmental impact. The local government (Balikpapan City, which administers Sungai Wain forest) played a key role in the conservation management planning and strongly supported the proposed alternative solution. But the provincial (East Kalimantan) government was not involved in the conservation plan and proceeded to build the road, in hindsight a mistake because the Pulau Balang road was primarily planned by the province. In contrast, the Balikpapan Bay Management Body, which was established by the international Project Pesisir and is responsible primarily for the area west of the proposed road, included the provincial government as one of the major stakeholders [3]. Unfortunately, the issue of the Pulau Balang road was completely omitted from their management planning. While the reasons remain unclear, it is likely that the issue of this planned road was considered too controversial, with stakeholders involved with the provincial government lobbying against raising the issue and other stakeholders avoiding argument about it so that political support for other conservation and sustainability agenda in Balikpapan Bay could be maintained. However, it is now clear that the latter strategy was a mistake, because after building the Pulau Balang road the coastal ecosystem is likely to collapse and all previous conservation expensive achievements of the Project Pesisir, together with several years of intensive work, are likely to be entirely wasted.

*Increase enforcement effort along existing roads through endangered species habitats*. The current Balikpapan government promised to increase enforcement along the planned Pulau Balang road by further empowering the Sungai Wain Protection Forest guards. It was further proposed that the area of the Sungai Wain Protection Forest would be extended north-east of the road. No increased enforcement was planned for the coastal mangrove ecosystems, but the overall protection of the area affected by the road construction was to be increased by allocating police and military facilities along the road within the proposed spatial plan. However, the government has so far paid lip service to these commitments, which were never captured in official documents. Observations suggest law enforcement has decreased along the existing Balikpapan-Samarinda provincial road, along the east boundaries of the same protected area (Sungai Wain Protection Forest), where the encroachment has already proceeded several kilometers from the road into the protected area. Furthermore, approximately 2 km^2^ of the protected area along the road was degazetted in the late 1990’s and an additional 14 km^2^ under encroachment was allocated as Community Forest in 2011, which is in practice a means of legalizing the ongoing illegal encroachment. At the same time, local government has violated laws that protect the coastal and riverside mangrove forests by issuing several permits for corporations to construct palm oil processing units and other industrial complexes in places that appear to benefit from the planned Pulau Balang road. All these examples indicate enhanced enforcement is unlikely to happen, encouraging other corporations and local people to disobey laws and further encroach into forested areas. In turn, ongoing illegal activities indirectly help the road development plans to proceed, because an argument is emerging that “the area is already disturbed and with no conservation value”. Thus, plans for greater enforcement effort along a planned road have little value, unless enforcement is initiated before the road construction begins and unless the commitment takes a written, mandatory form, preferably in the form of a government decree.

*Audit environmental and social impact assessments*. Environmental Impact Assessments (EIAs) of the Pulau Balang Bridge area and planned road project were conducted in 2006. The EIA’s recommendations are probably currently not applicable due to the time lag between assessment and initiation of construction, as well as technical changes to the project since the EIA was conducted. However, it is unlikely that a new EIA will be conducted. Fortunately, in Indonesia there is a legal mechanism that can enforce government transparency. Legal action by NGOs is often necessary to access documents, and has been brought against the provincial government for withholding the EIA and other documents concerned with the Pulau Balang road construction. Unfortunately, EIAs can be manipulated and the integrity of data collected in the 2006 Pulau Balang EIA appears questionable and recommendations unsound. An inaccurate EIA could be legally questioned but so far, most NGOs hesitate to sue the government. Ultimately, the main problem with EIAs is the absence of a legal framework to ensure developers comply with the recommendations. Indeed, many local environmental activists are highly hesitant to join EIA teams in fear of “greenwashing”, with their names and recommendations misused to allow a project to push ahead. Scientists familiar with the area of the Pulau Balang road and its wildlife were contacted for their opinions, with ecologists recommending an elevated road for wildlife corridors. To our knowledge, none of these recommendations were incorporated into the final project design, and as land was cleared for the first segment of the road in 2013, no allocation for wildlife corridors were apparent.


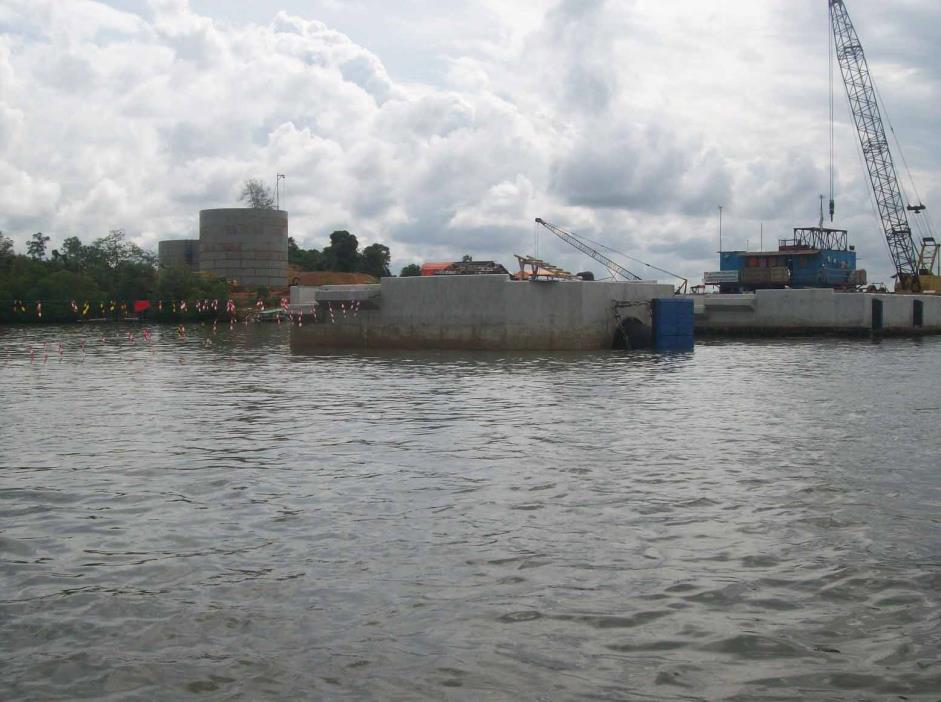


*Raise public awareness of environmental impacts of existing and proposed road projects*. Substantial effort was made to raise public awareness about the negative impact of the Pulau Balang project and about the benefits of alternative options. The numerous approaches included media releases, public lectures, TV and radio talks, Facebook groups, published brochures and leaflets, poster displays, organized field trips, petitions, and demonstrations on the streets. This awareness campaign, however, only slowed the project, but did bring significant changes in long term plans. As the campaign continued for several years, it gradually lost its “newsworthiness” in the media and among the public. Unfortunately, there are also indications that newspapers were manipulated by influential stakeholders not to provide space for the Pulau Balang controversy. Because most people will not be directly affected by the road development as it will be built in a uninhabited area, public interest waned as media coverage decreased. One possible weakness of the public campaign was its local scope, which mainly targeted district and provincial media and events, and was less strident at national and international levels. It was therefore perceived by most stakeholders as a local rather than a national environmental case (or an international case, due to proposed foreign investment). The profile of the case did increase somewhat after a resolution was passed by the Association for the Tropical Biology and Conservation (ATBC) to oppose the project [1]. However, many important decision makers, such as the National Development Planning Agency, the Ministry of Public Works, and the international investors, remained unconcerned about international pressure. Finally, another weakness of the campaign was a focus on raising public outcry to increase government pressure, instead of creating good political will, especially among the most influential stakeholders. For example, proponents of the Pulau Balang project not only achieved significant media coverage for their road development propaganda, but also made sure to present their case to influential politicians and investors. The proponents in the provincial government even offered investors permits to establish new palm oil plantations or coal mines to garner greater political support for the Pulau Balang project. We do not negate the importance of creating public pressure through media campaigns in order to achieve environmentally meaningful political decisions. Rather, we emphasize a highly sophisticated lobbying strategy is needed, which includes both public campaigns, as well as negotiations that target the most influential stakeholders behind closed doors.

In conclusion, the above-mentioned strategies did slow the rate of road development to some degree and delayed its negative impact on the wildlife in Balikpapan Bay. However, no long-term viable solutions have been enforced (although several alternatives and solutions have been proposed) and the tension between conservationists and developers remains unresolved. In hindsight, these four strategies could have been more successful if better planned. Ultimately, hidden agendas and lack of political will hampered wildlife protection in this case study. If current efforts continue to delay the development of the road until the next elections (on district, provincial and national level), it is still possible that the destruction of the ecosystem can be halted. Whatever the future outcome may be, Balikpapan Bay has proven an important case study. We hope that the lessons learned here will help other conservation practitioners mitigate the impacts of planned roads in other endangered mammal habitats in Southeast Asia.

**References**

1. Hance J (2010) Bridge Development in Kalimantan Threatens Rainforest, Mangroves, and Coral Reef. MONGABAY.COM. Available: http://news.mongabay.com/2010/0103-hance_pulau.html. Accessed 03 Aug 2014.
2. Fredriksson GM, de Kam M (1999) Strategic plan for the conservation of the Sungai Wain Protection Forest, East Kalimantan. The International Ministry of Forestry and Estate Crops.
3. Propinsi Kalimantan Timur, Proyek Pesisir Kalimantan Timur (2002) Rencana Strategis Pengelolaan Terpadu Teluk Balikpapan. Propinsi Kalimantan Timur.
