# Supplementary material for: Where and How Are Roads Endangering Mammals in Southeast Asia's Forests?
Source: PLoS One. 2014 Dec 18;9(12):e115376. doi: 10.1371/journal.pone.0115376 (PMC4270763; doi:10.1371/journal.pone.0115376)
Supplement: S6 Table — Supporting evidence from peer-reviewed articles and grey literature corroborating expert claims that roads contribute to forest conversion of habitats where endangered mammals occur in Southeast Asia. (DOCX) [file pone.0115376.s006.docx]

**Table S6*.*** Supporting evidence from peer-reviewed articles and grey literature corroborating expert claims that roads contribute to forest conversion of habitats where endangered mammals occur in Southeast Asia.

| **Country** | **Name of road/road network** | **Threatened endangered mammal habitats** | **Supporting evidence for threats from peer-reviewed articles and grey literature** |
| --- | --- | --- | --- |
| Cambodia | National Highway 4 | Kirirom and Bokor NP | Roadside forests vulnerable to illegal logging for firewood, charcoal and timber [1] |
|  | Provincial Road Network 76-141 | Eastern Plains Landscape* | Illegal timber stockpiles found along road bisecting Snuol [2] |
|  |  |  | Villagers paid to drag logs harvested from Snuol to the road side [3] |
|  | National Road 48 | Cardamom Mountains^ | Road has intensified illegal logging in neighbouring protected areas [4] |
| Indonesia |  |  |  |
| *Kalimantan* | Bontang-Sangata Road | Kutai NP | Road has spawned arterial roads that were utilized by both industrial timber companies and illegal loggers [5] |
|  |  |  | Road has intensified forest conversion to plantations [6] [7] |
|  |  |  | Forests along road now dominated by coal mines and oil palm plantations [8] |
|  | Balikpapan-Samarinda Road | Bukit Soeharto RF | Park has degraded due to road and expected to lose 100% of original forest cover by 2013 [9] |
|  | Logging road networks | Priority sites for Orangutan conservation# | Park buffer zones near logging roads suffered higher deforestation rates than those next to paved roads [10] |
| *Sumatra* | Logging road networks | Tiger conservation landscapes† | 49,020 km of logging roads has led to extensive forest loss and degradation [11] |
|  |  |  | Forests along logging roads prone to clearing and forest conversion by villagers for farmlands [12] |
| Lao PDR | Route Network 17A-3 | Nam Ha NBCA | Road has accelerated forest conversion for teak, rubber and sticky rice cultivation [13] |
| Malaysia |  |  |  |
| *East* | Kalabakan-Sapulut Road | FRs in Tawau and Pensiangan Districts | Road has contributed to overland illegal timber traffic out of East Kalimantan [14] |
|  | Logging road networks | FRs, Kelabit highlands | 300-km logging road by Samling Corporation has threatened to accelerate deforestation [15] |
|  | Access roads for dams | Murum, Danum and Pileran Valleys | Access road to Murum dam site has resulted in timber extraction from roadside forests [16] |
| Vietnam | Ho Chi Minh Highway | Protected areas§ | Highway has led to loss of habitat in protected areas beside it [17] |
|  | Roads in banteng habitats | Ea So, Yok Don and Krong Trai NR, Vinh Cuu NP | Roads has encouraged human settlement and elevated incidences of logging [18] [19] |
|  |  |  |  |

* Mondulkiri PF, Seima BCA, Lumphat, Snuol, Phnum Prech and Phnum Namlier WS

^ Phnum Samkos and Phnum Aural WS, Central Cardamom PF

\# Gunung Palung, Danau Sentarum/Bentung Kerihun, Tanjung Puting, Belantikan, Gunung Gajah/Berau/Kelai, Sebangau

† Kerinci Seblat NP, Tesso Nilo and Bukit Tigapuluh landscapes, Bukit Rambang Baling, Kuala Kampar-Kerumutan, Rimbo Panti-Batang Gadu, proHUsed Senepis-Buluhala Tiger National Park

§ Cuc Phuong and Phong Nha-Ke Bang NP, Vu Quang NR

NOTE: BCA = Biodiversity Conservation Area; FR = Forest Reserve; PA = Protected Area; PF = Protection Forest; NBCA = National Biodiversity Conservation Area; NP = National Park; NS = Nature Reserve; RF = Recreation Forest; WS = Wildlife Sanctuary

**References**

1. Food and Agriculture Organization (1998) Woodfuel Flow Study of Phnom Penh, Cambodia. Food and Agriculture Organization of the United Nations Regional Wood Energy Development Programme in Asia. Available: http://www.fao.org/docrep/x5667e/x5667e07.htm. Accessed 03 Aug 2014.
2. Société Générale de Surveillance (2005) Independent monitor: forest crime monitoring and reporting. Geneva: Société Générale de Surveillance.
3. Global Witness (1999) The Untouchables: Forest Crimes and the Concessionaires – can Cambodia Afford to keep them? London: Global Witness. 18 p.
4. Asian Development Bank (2005) Greater Mekong subregion biodiversity conservation initiative: Strategic framework and technical Assessment 2005-2014. Bangkok: Asian Development Bank. 221 p.
5. Jepson P, Momberg F, van Noord H (2002) A review of the efficacy of the protected area system of East Kalimantan province, Indonesia. Nature Area J 22: 28-42.
6. Vayda AP, Sahur A (1996) Bugis settlers in East Kalimantan’s Kutai National Park, their past and present and some possibilities for their future. Jakarta: Center for International Forestry Research Special Publication. 54 p.
7. World Bank (1998) Fire hazards, transboundary hazards, and sustainable forestry in East Asia and the Pacific. World Bank. Available: http://www.fire.uni-freiburg.de/course/coursere/id_coure_2.htm. Accessed 03 Aug 2014.
8. Setiawan A, Nugroho TS, Djuwantoko, Pudyatmoko S (2009) A survey of Miller’s Grizzled Surili*, Presbytis hosei canicurus*, in East Kalimantan, Indonesia. Primate Conserv 24: 139-143.
9. Harris NL, Petrova S, Stolle F, Brown S (2008) Indentifying optimal areas for REDD intervention: East Kalimantan, Indonesia as a case study. Environ Res Lett 3: 035006.
10. Curran L, Trigg N, McDonald AK, Astiani D, Hardiono YM et al. (2004) Lowland forest loss in protected areas in Indonesian Borneo. Science 303: 1000-1003.
11. Gaveau DLA, Epting J, Lyne O, Linkie M, Kumara I et al. (2009) Evaluating whether protected areas reduce tropical deforestation in Sumatra. J Biogeogr 36: 2165-2175.
12. Linkie M, Smith RJ, Leader-Williams N (2004) Mapping and predicting deforestation patterns in the lowlands of Sumatra. Biodivers Conserv 13: 1809-1818.
13. Butler R (2009) Illegal Hunting in Laos Takes a Toll on Wildlife. MONGABAY.COM. Available: http://news.mongabay.com/2009/0420-wildlife_trade_in_laos.html. Accessed 03 Aug 2014.
14. Obidzinski K, Andrianto A, Wijaya C (2007) Cross-border timber trade in Indonesia: critical or overstated problem? Forest governance lessons from Kalimantan. Int For Rev 9: 526-535.
15. Then S (2008) Concern over Logging Road into Bario Highland. The Star. Available: http://www.thestar.com.my/story.aspx?file=%2f2008%2f10%2f22%2fnation%2f20081022144217. Accessed 03 Aug 2014.
16. Then S (2009) Murum Dam: More than 100 Wildlife Species at Risk. The Star. Available: http://www.thestar.com.my/story.aspx?file=%2f2009%2f11%2f2%2fnation%2f20091102152540&sec=nation. Accessed 03 Aug 2014.
17. Gray DD (2006) Ho Chi Minh Trail Vietnam, from Soldier’s Road to Tourist Highway. Associated Press. Available: http://usatoday30.usatoday.com/travel/destinations/2005-07-18-ho-chi-minh_x.htm. Accessed 03 Aug 2014.
18. Nguyen MH (2009) The status of vulnerable gaur (*Bos gaurus*) and endangered banteng (*Bos javanicus*) in Ea So Nature Reserve and Yok Don and Cat Tien National Parks, Vietnam. Oryx 43: 129-135.
19. Pedrono M, Tuan HA, Chouteau P, Vallejo F (2009) Status and distribution of the endangered banteng (*Bos javinicus birmanicus*) in Vietnam: a conservation tragedy. Oryx 43: 618-625.
