# Supplementary material for: Where and How Are Roads Endangering Mammals in Southeast Asia's Forests?
Source: PLoS One. 2014 Dec 18;9(12):e115376. doi: 10.1371/journal.pone.0115376 (PMC4270763; doi:10.1371/journal.pone.0115376)
Supplement: S7 Table — Supporting evidence from peer-reviewed articles and grey literature corroborating expert claims that roads contribute to illegal hunting and trade of wildlife in Southeast Asia. (DOCX) [file pone.0115376.s007.docx]

**Table S7*.*** Supporting evidence from peer-reviewed articles and grey literature corroborating expert claims that roads contribute to illegal hunting and trade of wildlife in Southeast Asia.

| **Country** | **Name of road/road network** | **Threatened endangered mammal habitats** | **Supporting evidence for threats from peer-reviewed articles and grey literature** |
| --- | --- | --- | --- |
| Cambodia | National Highway 4 | Kirirom and Bokor NP | Road has provided access into Kirirom to illegally hunt mammals [1], [2] |
|  |  |  | Game is illegally sold along the road [3] |
|  | Provincial Road Network 76-141 | Eastern Plains Landscape* | Increased encounter rate of hunting signs along extension of road through the Seima [4] |
| Indonesia |  |  |  |
| *Sumatra* | Sanggi-Bengkunat/Krui Liwa Roads | Bukit Barisan Selatan NP | Roads have provided access to poachers, who are removing Sumatran tigers from the park each year [5] |
|  | Blangkejeren-Kutacane Road | Gunung Leuser NP | Roads have contributed to hunting in park (Singleton et al. 2004), and declines of Sumatran Orangutans [6] |
|  | Logging road networks | Tiger conservation landscapes† | Logging road networks have contributed poacher access into Sumatran Tiger habitats [7] |
|  |  |  | Logging highways have increased levels of human and Sumatran Tiger conflicts [8] |
| Lao PDR | Route 9 | Phou Xang He and Dong Phou Vieng NBCAs | Road upgrade has increased the risk of cross-border trafficking of mammals [9] |
|  | Route Network 12-1E-8 | Nakai-Nam Theun NBCA | Road has threatened Saola and Douc Langurs, particularly through poaching for illegal trade [10]. |
| Malaysia |  |  |  |
| *East* | Kalabakan-Sapulut Road | FRs in Tawau and Pensiangan Districts | One Sumatran rhinoceros individual poached along road after it became habituated to presence of people [11] |
|  | Logging road networks | FRs, Kelabit highlands | Logging roads from Pan Borneo Highway allowed poachers to access Bornean Pygmy Elephant habitats [12] |
|  |  |  | Main Line West logging road has increased poaching threat to Bornean Pygmy Elephants [13] |
| *Peninsular* | Federal Route 4 | Royal Belum State Park, Temengor FR | Roadside patrols removed snares, rescued a snared tiger and arrested poachers [14], [15] |
|  |  |  | More snares per unit survey effort were detected closer to road than forest interior [16] |
|  | Federal Route 8 | Tamana Negara NP, Titiwangsa Main Range | Road re-alignment to within 2 km of national park has provided greater access for poachers [17] |
|  | State Route T156 | Tembat, Petuang and Hulu Telemong FRs | See Results |
| Myanmar | Wildlife trade route network | All mammal habitats in Myanmar | See Results |
|  | Ledo road | Hukaung Valley WS | Road has provided unrestricted access to poachers, who actively supply wild meat to local markets [18] |
| Vietnam | Ho Chi Minh Highway | Protected areas§ | Highway has led to hunting in adjacent protected areas [19], especially to Saola populations [20] |
|  |  |  | Arterial roads branching from highway function as conduits for illegal wildlife trade [21], [22] |
|  | Roads in banteng habitats | Ea So, Yok Don and Krong Trai NR, Vinh Cuu NP | Roads have increased extirpation risk of Banteng due to increased accessibility for hunters [23] |
|  | Roads in | Cat Tien NP | Roads have provided access to poachers targeting mammals such as the Javan rhino [24] |
|  |  |  |  |

* Mondulkiri PF, Seima PF, Lumphat, Snuol, Phnum Prech and Phnum Namlier WS

† Kerinci Seblat NP, Tesso Nilo and Bukit Tigapuluh landscapes, Bukit Rambang Baling, Kuala Kampar-Kerumutan, Rimbo Panti-Batang Gadu, proHUsed Senepis-Buluhala Tiger National Park

§ Cuc Phuong and Phong Nha-Ke Bang NP, Vu Quang NR

NOTE: BCA = Biodiversity Conservation Area; FR = Forest Reserve; PA = Protected Area; PF = Protection Forest; NBCA = National Biodiversity Conservation Area; NP = National Park; NS = Nature Reserve; RF = Recreation Forest; WS = Wildlife Sanctuary

**References**

1. Khim L, Taylor-Hunt D, Monirak M, Sarenn K, Savuth U (1995) Kirirom General Survey. Phnom Penh: Ministry of Environment, Department of Nature Conservation and Protection.
2. Asian Development Bank (2005) Greater Mekong subregion biodiversity conservation initiative: Strategic framework and technical Assessment 2005-2014. Bangkok: Asian Development Bank. 221 p.
3. Palmer B, Martin S (2008) The Rough Guide to Cambodia. London: Rough Guides Limited. 352 p.
4. Wildlife Conservation Society (Cambodia Programme) (2004 – 2012) Unpublished data.
5. O’Brien TG, Kinnaird MF, Wibisono HT (2003) Crouching tigers, hidden prey: Sumatran populations in a tropical forest landscape. Anim Conserv 6: 131-139.
6. van Schaik CP, Monk KA, Robertson JMY (2001) Dramatic decline in Orang-utan numbers in the Leuser Ecosystems, Northern Sumatra. Oryx 35: 14-25.
7. Gillison AN (2001) Vegetation survey and habitat assessment of the Tesso Nilo forest complex. Yungaburra: Center for Biodiversity Management. 68 p.
8. Eyes on the Forest (2008) Asia Pulp and Paper threatens Senepis forest, Sumatran tiger habitat, and global climate. Riau: Eyes on the Forest 15 p.
9. Asian Development Bank (2008) Lao People’s Democratic Republic and Socialist Republic of Vietnam: Greater Mekong subregion: east-west corridor project, Performance Evaluation Report. Available: http://www.adb.org/sites/default/files/projdocs/2008/41353-REG-TAR.pdf. Accessed 03 Aug 2014.
10. Timmins RJ, Duckworth JW (2004) Status and conservation of Douc Langurs (*Pygathrix nemaeus*) in Laos. Int J Primatol 20: 469-489.
11. Unet R (2009) Concern over Sapulut-Kalabakan Highway. Available: http://sabahmajujaya.blogspot.com/2009/03/concern-over-sapulut-kalabakan-highway.html. Accessed 03 Aug 2014.
12. J Payne Pers. Comm.
13. ST Wong, Pers. Comm.
14. New Straits Times (05 Oct 2009) Trapped Tiger Saved, but More Patrols Needed. Available: http://bigcatrescue.blogspot.com/2009/10/tiger-rescue-points-to-urgent-need-for.html. Accessed 03 Aug 2014.
15. Clements R, Rayan MD, Ahmad Zafir AW, Venkataraman A, Alfred R et al. (2010) Trio under threat: can we secure the future of rhinos, elephants and tigers in Malaysia. Biodivers Conserv 19: 1115-1136.
16. MD Rayan, unpublished data.
17. Sharma DDD (2009) Taman Negara Threatened by New Road. Malaysiankini. Available: http://www.malaysiakini.com/letters/103252. Accessed 03 Aug 2014.
18. Rabinowitz A (2004) A question of balance. Natl Geogr 205: 98-117.
19. Gray DD (2006) Ho Chi Minh Trail Vietnam, from Soldier’s Road to Tourist Highway. Associated Press. Available: http://usatoday30.usatoday.com/travel/destinations/2005-07-18-ho-chi-minh_x.htm. Accessed 03 Aug 2014.
20. Stone R (2006) The Saola’s last stand. Science 314: 1380-1383.
21. World Bank (2005) Going, going, gone: The illegal trade in wildlife in East and Southeast Asia. Washington DC: Environment and Social Development Department, East Asia and Pacific Region. 32 p.
22. Shepherd CR, Compton J, Warne S (2007) Transport Infrastructure and Wildlife Trade Conduits in the GMS: Regulating Illegal and Unsustainable Wildlife Trade. Available: http://www.traffic.org/non-traffic/bci-paper.pdf. Accessed 03 Aug 2014.
23. Nguyen MH (2009) The status of vulnerable gaur (*Bos gaurus*) and endangered banteng (*Bos javanicus*) in Ea So Nature Reserve and Yok Don and Cat Tien National Parks, Vietnam. Oryx 43: 129-135.
24. Polet G, Ling S (2004) Protecting mammal diversity: opportunities and constraints for pragmatic conservation management in Cat Tien National Park, Vietnam Oryx 38: 186-196.
